# SMRT: Randomized Data Transformation for Cancer Subtyping and Big Data Analysis

**DOI:** 10.3389/fonc.2021.725133

**Published:** 2021-10-20

**Authors:** Hung Nguyen, Duc Tran, Bang Tran, Monikrishna Roy, Adam Cassell, Sergiu Dascalu, Sorin Draghici, Tin Nguyen

**Affiliations:** ^1^ Department of Computer Science and Engineering, University of Nevada Reno, Reno, NV, United States; ^2^ Department of Computer Science, Wayne State University, Detroit, MI, United States

**Keywords:** cancer subtyping, multi-omics integration, web application, CRAN package, survival analysis

## Abstract

Cancer is an umbrella term that includes a range of disorders, from those that are fast-growing and lethal to indolent lesions with low or delayed potential for progression to death. The treatment options, as well as treatment success, are highly dependent on the correct subtyping of individual patients. With the advancement of high-throughput platforms, we have the opportunity to differentiate among cancer subtypes from a holistic perspective that takes into consideration phenomena at different molecular levels (mRNA, methylation, etc.). This demands powerful integrative methods to leverage large multi-omics datasets for a better subtyping. Here we introduce Subtyping Multi-omics using a Randomized Transformation (SMRT), a new method for multi-omics integration and cancer subtyping. SMRT offers the following advantages over existing approaches: (i) the scalable analysis pipeline allows researchers to integrate multi-omics data and analyze hundreds of thousands of samples in minutes, (ii) the ability to integrate data types with different numbers of patients, (iii) the ability to analyze un-matched data of different types, and (iv) the ability to offer users a convenient data analysis pipeline through a web application. We also improve the efficiency of our ensemble-based, perturbation clustering to support analysis on machines with memory constraints. In an extensive analysis, we compare SMRT with eight state-of-the-art subtyping methods using 37 TCGA and two METABRIC datasets comprising a total of almost 12,000 patient samples from 28 different types of cancer. We also performed a number of simulation studies. We demonstrate that SMRT outperforms other methods in identifying subtypes with significantly different survival profiles. In addition, SMRT is extremely fast, being able to analyze hundreds of thousands of samples in minutes. The web application is available at http://SMRT.tinnguyen-lab.com. The R package will be deposited to CRAN as part of our PINSPlus software suite.

## 1 Introduction

Since cancer is a heterogeneous disease, the correct identification of cancer subtypes is essential for accurate prognosis and improved treatment. With the advancement of high-throughput platforms, subtyping methods have shifted toward multi-omics integration in order to differentiate between subtypes from a holistic perspective that takes into consideration phenomena at different molecular levels (mRNA, methylation, etc.). Vast amounts of molecular data have accumulated in public repositories, including The Cancer Genome Atlas datasets (TCGA) (1), Genomic Data Commons Data Portal (GDC) (2), Molecular Taxonomy of Breast Cancer International Consortium (METABRIC) (3), and UK Biobank (4). This demands powerful yet fast analysis methods to leverage large multi-omics datasets for a more accurate subtype discovery.

Current approaches for multi-omics integration and cancer subtyping can be categorized into four categories based on their integration strategy. The first strategy is to concatenate different types of data into a single matrix and then partition the patients using the concatenated data. For example, users can normalize and concatenate multiple data types (e.g., mRNA, methylation, miRNA, etc.) into one single matrix and then apply well-known methods developed for single-omics analysis, such as ConsensusClusterPlus (5), to determine the subtypes. Such approaches are simple and computationally efficient. However, they do not account for data heterogeneity, e.g., different data types might have different scales, dimensions and might require different normalization procedures.

The second strategy is to model the multi-omics data as a mixture of statistical models. Methods in this category include LRACluster (6), rMKL-LPP (7), iClusterPlus (8), iClusterBayes (9), OTRIMLE (10), SBC (11), BCC (12), MID (13), JIVE (14), MCIA (15), moCluster (16), and sMBPLS (17). These methods typically maximize a joint likelihood function to determine the model parameters and the subtypes. Though statistically sound, these methods need to estimate a large number of parameters that often lead to overfitting and high computational complexity. Therefore, an added step of gene filtering or data transformation is often applied before the statistical analysis.

The third strategy is to project all data types into a joint latent space. A common technique used for this strategy is non-negative matrix factorization. Methods in this category include MvNMF (18), MultiNMF (19), IntNMF (20), iNMF (21), jointNMF (22). Another method is MCCA (23) that performs correlation analysis and then concatenates the correlation matrices into one single matrix. After projecting the data onto a joint space, cluster analysis is performed to determine the final subtypes. Similar to the second strategy, methods in this category often have excessive computational complexity and cannot be applied on the whole genome-scale. Therefore, gene filtering is a necessary step in the data processing.

The fourth strategy is also called similarity-based strategy. Methods in this category include SNF (24), PSDF (25), PFA (26), IS-Kmeans (27), NEMO (28), PINS (29, 30), SCFA (31), and CIMLR (32). These methods first compute a pair-wise connectivity matrix for each data type, that represents the similarity/connectivity between patients. The connectivity matrices are then fused onto a single similarity matrix that can be used for the final clustering. Although powerful, the similarity matrix requires a quadratic memory space. This is problematic when the number of samples increases. As we will demonstrate in our analysis, these methods cannot analyze data with tens of thousands of samples.

Here we introduce Subtyping Multi-omics using a Randomized Transformation (SMRT), a new method for cancer subtyping and big data analysis. This method offers important advantages over existing software: (i) it allows researchers to analyze hundreds of thousands of samples in minutes, (ii) it can integrate data types with different numbers of patients, (iii) the ability to integrate and analyze un-matched data of different types, and (iv) the web application offers a convenient data analysis pipeline. We also improve the efficiency of our ensemble-based, perturbation clustering to support analysis on machines with memory constraints. Our extensive analysis on 37 TCGA and two METABRIC datasets shows that SMRT is more accurate than state-of-the-art subtyping methods in identifying subtypes with significantly different survival profiles. In addition, our simulations with big data show that SMRT is fast and many-fold more scalable than existing methods. Specifically, SMRT is able to analyze hundreds of thousands of samples in minutes.

## 2 Materials and Methods

### 2.1 The SMRT Pipeline

**Figure 1:**
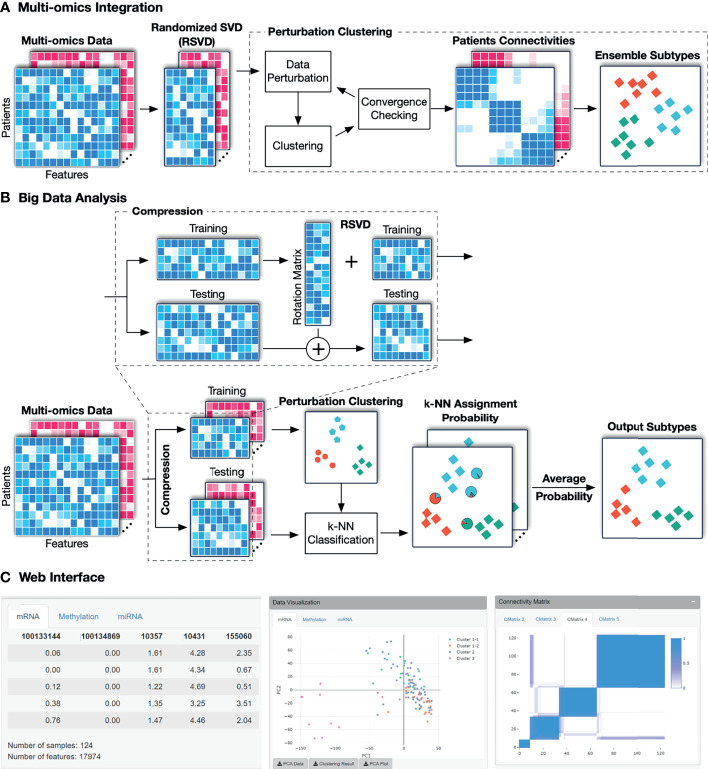
The overall workflow of SMRT. **(A)** Analysis pipeline for data with moderate size. First, SMRT projects each data type to a lower-dimensional space using randomized singular value decomposition (RSVD). Next, it performs a perturbation clustering to determine the subtypes, and to build a pair-wise patient connectivity for each data type. Finally, it merges the connectivity matrices onto a single similarity matrix and then determines the final subtypes using a cluster ensemble. The output is the clustering results for each data type, as well as the results after the multi-omics data integration. **(B)** Analysis pipeline for big data. SMRT first splits the data into two different sets: a sampled and a propagated set. The method first determines the subtypes using the sampled set and then assigns the patients from the propagated set to subtypes identified using the sampled set. The sampled data is partitioned using the pipeline described assignments for samples in the propagated set are determined by averaging the probabilities from all k-NN models. **(C)** An example of the subtypes discovered by the SMRT web service for the KIRC dataset. The left panel shows a preview of the uploaded data. The middle panel shows the visualization of the discoveredSMRT web service for the KIRC dataset. The left panel shows a preview of the uploaded data. The middle panel shows the visualization of the discovered subtypes and export functions. The right panel shows patient connectivity matrices for each data type.

The overall workflow of SMRT is presented in **Figure 1**. This workflow offers two different analysis pipelines for big data and data with a moderate size. In the first case, given a multi-omics dataset with a moderate size (e.g., less than 2,000 samples), SMRT performs subtyping as follows. It first projects each data type onto a lower-dimensional space using randomized singular value decomposition (RSVD) and then performs a perturbation clustering (PINS) (29, 30) to determine the subtypes within each data level. It also builds a pair-wise connectivity matrix for each data type that represents the connectivity between patients red (See **Supplementary Section 5** for the differences between SMRT and PINS). Next, the method combines the connectivity matrices into a single similarity matrix and then determines the final subtypes using an ensemble of multiple similarity-based methods. In the second case, when the data has more than 2,000 samples, SMRT splits the data into two different sets of patients: a sampled set and a propagated set. It then performs the subtyping on the sampled set and then assigns the patients from the propagated set to the identified subtypes. Note that the number 2,000 is chosen to balance between the accuracy and time complexity of the method. This moderate number of samples allows SMRT to perform a fast and accurate analysis in limited memory (see **Supplementary Section 3**). Our simulation studies show that the results do not change when we vary this number. However, users are free to change this parameter when using the R package. Below is the description of each of these analysis modules.

### 2.2 Dimension Reduction Using Randomized Singular Value Decomposition

The goal of this step is to project the multi-omics data into a lower-dimensional space using randomized singular value decomposition (RSVD). For data with hundreds of thousands of dimensions (e.g., Illumina 450k), this step substantially reduces the required computational power while maintaining the clustering accuracy. Let us denote *X* ∈ ℝ*^n^* ^×^ *^m^* as the input matrix, where *n* is the number of samples/patients, and *m* is the number of genes/features. Briefly, the RSVD method starts by generating a random projection matrix *P* ∈ ℝ*^m^* ^×^ *^r^* from a standard normal distribution where *r* ≪ *m*. It then projects *X* ∈ ℝ*^n^* ^×^ *^m^* to the column space of *P* to get a matrix *Z* such that *Z* = *XP*. Due to the random projection, *Z* and *X* will have approximately the same dominant columns (features). Now, we can obtain the orthogonalized matrix *Q* of *Z* by using *QR* decomposition, where *Q* has the same size as *Z* of *n* × *r*. In the next step, the method projects *X* into a smaller space to get a matrix *Y* ∈ ℝ*^r^* ^×^ *^m^* such that *Y* = *Q^^T^* *X and then computes singular value decomposition (SVD) of *Y* as *Y* = UΣ*V* ^*^ using the traditional SVD method (33). *U* and *V* matrices only keep at most *r* eigenvectors so the size of *U* is *r* × *r* and the size of V* is *m* × *r*. Finally, the low rank rotated data of the original matrix *X* can be computed using: *X*′ = *XV* ^*^.

In practice, RSVD is faster and requires less memory than the traditional SVD. To further speed up our approach, we implement a parallel version of RSVD that can efficiently utilize multiple cores available in modern processors. Note that when the input data is large (e.g., more than 2,000 samples), we do not perform RSVD on the whole input. Instead, we split the data into two sets of patients: a sampled set and a propagated set. We first perform RSVD on the sampled set, and then project the original data matrix (both sampled and propagated set) to the subspace of the *sampled set* by multiplying it with the rotation matrix obtained from the RSVD for the sampled set. This implementation allows us to perform SVD in at most a few seconds, even for datasets with hundreds of thousands of samples and features.

The output of this module is multiple matrices – one per data type. In each matrix, the rows represent patients while the columns represent the principal components (PCA). These matrices will serve as input of the next module: perturbation clustering that will be described in the next section. This will compute the perturbed connectivity matrices and determine the subtypes.

### 2.3 Subtype Discovery Using One Data Type

Given a single data type, SMRT utilizes our previously developed perturbation clustering (PINS) (29, 30) to partition the data. Briefly, we perturb the data (by adding Gaussian noise) and repeatedly partition the patients (using k-means by default). For each partitioning, we build a pair-wise connectivity matrix of 0’s and 1’s in which 1 means that the two patients belong to the same cluster, and 0 otherwise. By perturbing and clustering the data multiple times, we obtain multiple connectivity matrices that represent how stable the connectivity between patient pairs. Finally, we choose the partitioning that is the most stable to data perturbation. This algorithm automatically determines the number of clusters and patient subgroups.

When the number of samples is large, the perturbation clustering becomes slow and memory-inefficient. The perturbation clustering algorithm relies on the pair-wise connectivity of size *n* × *n* for clustering (*n* is the number of patients). The time and space complexity (running time and memory usage) of this method increase quadratically when the number of samples increases. Therefore, when the number of samples is large (by default setting, when *n* > 2,000), we perform a sub-sampling process over the original data to obtain a subset of 2,000 patients/samples. Next, we transform the data into a lower-dimensional space, and use the perturbation clustering to partition these patients. After this step, each of the 2,000 patients has a subtype. Let us refer to this selected set of 2,000 patients as the *sampled set*. The next step is to determine the subtypes for the rest of the patients, called the *propagated set*. For this purpose, we use the fast k-nearest neighbor searching algorithms (FKNN) algorithm (34, 35) to assign each patient from the propagated set to one of the subtypes in the sampled set. Briefly, the FKNN method calculates the distance between the new patient to the *k* nearest patients in the sampled set. Next, the FKNN method classifies the new patient using vote counting (i.e., it chooses the subtype with the most patients among the *k* neighbors). By default, *k* is determined using the Elbow method on the sampled set using 5-fold cross-validation. The sampled set is divided randomly into 5 equally smaller sets. In each round, the combination of 4 sets is used as the training set, and the other is used as the validation set for the KNN algorithm with *k* ranges from 5 to a maximum of 50. The *k* that yields the lowest average classification error rate will be used as the optimal *k*. However, users are also free to modify the value of this parameter. **Supplementary Section 6** provides more details on the performance of using the Elbow method *versus* using a fixed number of *k*.

One note of caution is that the number of dimensions of the data can be high, thus slowing the process of distance calculation and neighbor finding. Therefore, instead of calculating the distance between patients in the original space, we calculate the distance between patients in the principal component (PC) space of the sampled set. As described above, we project the original data matrix (both sampled and propagated set) to the subspace of the *sampled set* by multiplying it with the projection matrix obtained from the RSVD for the sampled set. After this transformation, the pair-wise distance between patients will be calculated in the new space with a much lower number of dimensions.

### 2.4 Subtype Discovery Using Multi-Omics Data

When the number of samples is small (by default, when *n* ≤ 2,000), we utilize an ensemble strategy to partition the patients. The method first clusters each data type (using the algorithm described in Section 2.3) and constructs the perturbed connectivity matrices. It then merges the connectivity matrices of all data types to a single similarity matrix that represents the similarity between patients across all data types by averaging the connectivity values for each pair of samples. Next, to cluster the similarity matrix, it uses several similarity-based algorithms, including hierarchical clustering, partitioning around medoids (36), and dynamic tree cut (37) and then chooses the partitioning that agrees the most with the partitioning of individual data types. This ensemble strategy ensures that the identified subtypes are consistent across all data types and are robust against the choice of clustering algorithms.

When the number of samples is large (by default, when n > 2,000), we perform a sub-sampling and classifying procedure that is similar to the algorithm described in the Section 2.3. The difference here is that multiple data types are involved. First, we randomly select 2,000 samples/patients and then apply the multi-omics algorithm described above to partition the selected samples. We refer to this selected set of 2,000 patients as the *sampled set* and the remaining patients as the *propagated set*. The next task is to determine the subtypes of patients in the propagated set. Given a patient in the propagated set, we perform the FKNN procedure for each data type to obtain the probability that it belongs to each subtype using the labels obtained from the nearest neighbors. The final probabilities are calculated by averaging the probabilities across all data types. Finally, we classify the patient to the subtype that has the highest probability. This strategy is also applied when integrating multi-omics data whose each data type has different number of samples. Here the sampled set will be the set of patients (by default, maximum 2,000 patients) that have data in all data types, and the remaining patients will be in the propagated set.

### 2.5 The SMRT Web Interface

The web application is publicly available at http://SMRT.tinnguyen-lab.com. The website is built using the R Shiny framework (38). Shiny is an R package that allows developers to directly build an interactive web interface using the R programming language. We use the web interface to forward data and requests from users to the new SMRT method to perform data integration and clustering. Because of the efficiency of the SMRT method, the website is able to return the results in minutes even for datasets with hundreds of thousands of samples.

Analysis using the web application is simple and straightforward. Users can either upload expression data in .csv files or a single .rds file using the upload function on the left panel. Each data type is presented as a matrix in which rows represent samples and columns represent genes/features. SMRT can automatically determine the number of subtypes. It does not require any extra configuration or parameters to perform the analysis. See **Supplementary Section 4** and **Figures S6**, **S7** for a more detailed description of the web application.

## 3 Results

To assess the performance of SMRT, we perform an extensive analysis using 39 cancer datasets and simulated data. First, we demonstrate that SMRT is able to identify cancer subtypes with significantly different survival profiles. Second, we provide an in-depth analysis for a Glioma dataset. Finally, we illustrate the scalability of SMRT by analyzing simulated datasets with hundreds of thousands of samples. We also provide a comparative analysis between subtypes discovered by SMRT and those of PAM50 classifier on three Breast cancer datasets (TCGA-BRCA, METABRIC_Discovery, and METABRIC_Validation) in **Supplementary Section 7**.

### 3.1 Experimental Studies Using 39 Cancer Datasets

In this article, we analyze 37 TCGA and 2 METABRIC datasets. For TCGA datasets, we downloaded the matched mRNA, DNA methylation, and miRNA expression data from the TCGA data portal. For the METABRIC datasets, we were able to obtain matched mRNA and copy number variation data from the European Genome-Phenome Archive. We also downloaded clinical data and survival information of each patient, which will be used to assess the performance of the subtyping methods. **Supplementary Tables 1**, **2** provide more details of the datasets.

We compare SMRT with eight state-of-the-art subtyping algorithms: SNF (24), CIMLR (32), NEMO (28), moCluster (16), iClusterBayes (9), LRACluster (6), MCCA (23), and IntNMF (20). The following packages were used in our comparison: SNFtool v2.3.0 on CRAN for SNF, CIMLR v1.0.0 at https://github.com/danro9685/CIMLR for CIMLR, NEMO v0.1.0 at https://github.com/Shamir-Lab/NEMO for NEMO, mogsa v1.16.0 on Bioconductor for moCluster, iClusterPlus on Bioconductor v1.18.0 for iClusterBayes, LRACluster v1.18.0 at http://bioinfo.au.tsinghua.edu.cn/member/jgu/lracluster/ for LRACluster, PMA v1.2.1 on CRAN for MCCA, and IntNMF on CRAN v1.2.0 for IntNMF. When the number of dimensions exceeded 2,000, we used only the top 2,000 variables with the largest variance for iClusterBayes, IntNMF, and MCCA, because these methods cannot analyze the data on the whole-genome scale. For all methods, we used default parameters and let all methods automatically determine the optimal number of clusters. For MCCA, which is not a clustering method itself, we follow the implementation at https://github.com/Shamir-Lab/Multi-Omics-Cancer-Benchmark for cluster analysis.

Using each method, we partition the patients in each dataset, and then assess the survival difference of the discovered patient groups using Cox regression (39). Overall survival data is used for TCGA datasets and Disease-free survival data is used for METABRIC datasets. **Table 1** shows the Cox p-values obtained from each dataset and method (See **Supplementary Section 9**, **Figures S10**–**S17** for the Kaplan-Meier survival curves for each dataset). There are seven datasets in which no method is able to identify subtypes with significant Cox p-values. For the remaining 32 datasets, SMRT has significant p-values in 28 datasets, whereas NEMO has significant p-values in 19 datasets and all other methods have significant p-values in 15 datasets or less. SMRT has the most significant p-values in 12 datasets out of those 28 datasets, while SNF, CIMLR, NEMO, moCluster, iClusterBayes, LRACluster, MCCA, and IntNMF have the most significant p-values in 0, 3, 8, 4, 2, 0, 1, and 2 datasets, respectively.

**Table 1:** Cox p-values of subtypes discovered by SNF, CIMLR, NEMO, moCluster, iClusterBayes (iCB), LRACluster (LRA), MCCA, IntNMF, and SMRT for 37 TCGA datasets and two METABRIC breast cancer datasets (M_Discovery and M_Validation).

Dataset	SNF	CIMLR	NEMO	moCluster	iCB	LRA	MCCA	IntNMF	SMRT
1. ACC	4.34e-05	3.96e-01	2.07e-04	2.63e-09	4.26e-03	2.46e-03	1.24e-08	6.11e-03	1.33e-02
2. BLCA	1.09e-01	3.09e-01	6.74e-02	3.13e-01	4.95e-01	7.42e-02	3.57e-01	3.43e-02	1.95e-02
3. BRCA	1.19e-01	4.95e-03	2.93e-02	2.58e-01	3.07e-02	3.90e-01	3.80e-04	2.53e-01	1.96e-03
4. CESC	5.10e-01	1.90e-01	3.33e-01	1.81e-01	1.69e-01	2.90e-01	6.69e-01	8.89e-01	2.95e-02
5. CHOL	5.72e-01	3.35e-01	3.02e-01	5.17e-01	6.51e-01	6.93e-01	4.50e-01	9.63e-01	3.01e-02
6. COAD	1.28e-01	2.52e-01	6.76e-01	3.73e-01	6.47e-01	5.05e-01	6.20e-01	5.35e-01	1.44e-03
7. COADREAD	6.60e-01	1.35e-01	8.11e-01	4.72e-02	2.55e-01	7.47e-01	7.87e-01	4.76e-01	2.89e-03
8. DLBC	7.55e-01	7.44e-01	3.53e-01	9.82e-01	7.42e-01	8.94e-01	8.15e-01	7.28e-01	4.74e-01
9. ESCA	3.92e-01	3.91e-01	3.92e-01	5.01e-01	3.75e-01	1.71e-01	2.25e-01	4.90e-01	3.30e-01
10. GBM	2.08e-02	8.11e-02	1.49e-04	5.12e-01	1.24e-01	5.37e-01	3.69e-01	7.04e-01	8.75e-05
11. GBMLGG	4.75e-14	6.36e-10	2.31e-17	6.46e-16	8.66e-12	8.04e-14	3.83e-07	1.25e-10	7.48e-17
12. HNSC	3.66e-01	6.19e-01	7.41e-05	2.44e-01	1.42e-01	3.27e-01	9.88e-01	1.55e-01	4.56e-02
13. KICH	7.01e-01	4.63e-01	8.14e-14	0.00e+00	4.03e-01	2.10e-01	8.08e-01	6.61e-01	2.77e-02
14. KIPAN	2.11e-07	9.84e-05	4.81e-08	4.04e-13	2.16e-08	4.21e-08	3.82e-03	4.36e-04	1.16e-11
15. KIRC	6.91e-01	9.79e-01	2.46e-01	1.76e-01	6.70e-01	1.76e-01	1.32e-01	7.29e-01	5.98e-05
16. KIRP	5.33e-03	1.85e-02	8.42e-18	1.00e+00	4.60e-02	5.97e-03	2.49e-02	1.93e-01	1.15e-09
17. LAML	1.73e-03	1.24e-02	5.14e-04	7.00e-01	9.38e-01	1.19e-01	1.75e-02	7.78e-02	8.72e-04
18. LGG	1.60e-14	7.14e-15	1.17e-17	3.52e-01	6.08e-03	1.01e-01	1.16e-09	4.04e-02	4.26e-15
19. LIHC	3.34e-01	1.28e-01	1.09e-03	8.25e-01	2.57e-01	2.93e-01	5.04e-01	8.80e-01	7.04e-01
20. LUAD	5.01e-01	3.73e-01	7.51e-03	5.92e-01	2.55e-02	1.49e-01	2.08e-01	8.21e-03	4.66e-01
21. LUSC	8.71e-02	3.91e-02	1.32e-01	7.04e-01	5.11e-01	9.05e-01	2.88e-01	6.75e-01	8.37e-03
22. MESO	4.24e-04	1.72e-02	7.94e-04	7.29e-02	8.66e-05	2.77e-01	5.53e-04	3.85e-04	7.34e-04
23. OV	4.45e-01	5.88e-01	6.95e-01	9.73e-01	4.35e-01	6.47e-01	7.78e-01	9.60e-01	6.81e-01
24. PAAD	7.36e-04	2.03e-03	1.44e-03	2.96e-03	4.19e-03	4.86e-04	3.18e-01	3.45e-02	2.73e-04
25. PCPG	3.32e-01	4.57e-01	2.57e-01	3.11e-01	3.39e-01	1.41e-01	6.63e-01	7.67e-01	8.66e-01
26. PRAD	4.75e-01	6.95e-01	6.61e-01	9.56e-01	3.73e-01	4.97e-01	7.07e-01	3.90e-01	3.49e-01
27. READ	7.62e-01	3.35e-01	6.27e-01	1.00e+00	5.68e-01	2.72e-01	3.53e-01	3.41e-01	2.35e-02
28. SARC	4.37e-02	5.58e-02	7.23e-02	3.37e-02	3.07e-01	6.36e-01	9.54e-02	2.83e-01	3.03e-02
29. SKCM	4.78e-01	7.54e-05	6.37e-04	4.30e-03	4.67e-03	3.92e-02	1.90e-01	1.48e-03	1.05e-01
30. STAD	4.07e-02	5.11e-01	1.02e-01	4.83e-01	6.25e-01	3.08e-01	3.16e-01	5.55e-01	1.86e-04
31. STES	1.57e-01	3.41e-02	1.18e-01	4.97e-01	4.13e-03	5.92e-01	6.35e-02	8.45e-02	1.51e-02
32. TGCT	8.38e-01	8.39e-01	8.38e-01	5.89e-01	2.96e-01	3.74e-01	5.65e-01	5.41e-01	5.31e-01
33. THCA	6.20e-01	8.62e-03	3.87e-02	5.11e-01	7.42e-01	5.51e-01	3.87e-01	1.75e-02	8.82e-02
34. THYM	9.69e-02	1.15e-01	7.11e-02	8.89e-05	7.06e-02	5.96e-01	5.47e-02	1.38e-01	1.33e-02
35. UCEC	1.81e-02	1.70e-01	1.64e-01	6.88e-01	1.65e-01	8.61e-01	1.58e-02	3.02e-03	4.83e-03
36. UCS	8.59e-01	3.59e-01	7.16e-01	1.68e-01	8.76e-01	8.34e-01	5.85e-01	6.27e-01	4.26e-01
37. UVM	1.67e-04	5.80e-04	1.67e-04	5.50e-01	9.19e-02	4.92e-03	2.06e-04	2.20e-05	6.43e-03
38. M_Discovery	2.26e-05	3.15e-12	1.16e-11	2.87e-01	9.16e-01	4.32e-06	4.59e-10	2.01e-07	3.25e-10
39. M_Validation	1.04e-02	4.68e-06	2.75e-07	1.57e-01	1.97e-01	1.28e-01	7.46e-04	9.16e-04	2.66e-05
#Significant	15	15	19	9	11	8	12	14	28

Cells highlighted in yellow have significant Cox p-values at the threshold of 5%. Cells highlighted in green have the most significant Cox p-value in their respective rows. No methods were able to yield subtypes with significantly different survival in 7 data sets (shown with red fonts). SMRT yields subtypes with significantly different survival profiles in 28 out of the 39 datasets. In 12 such datasets, SMRT also p-values more significant than any of those provided by the other eight methods.

**Figure 2:**
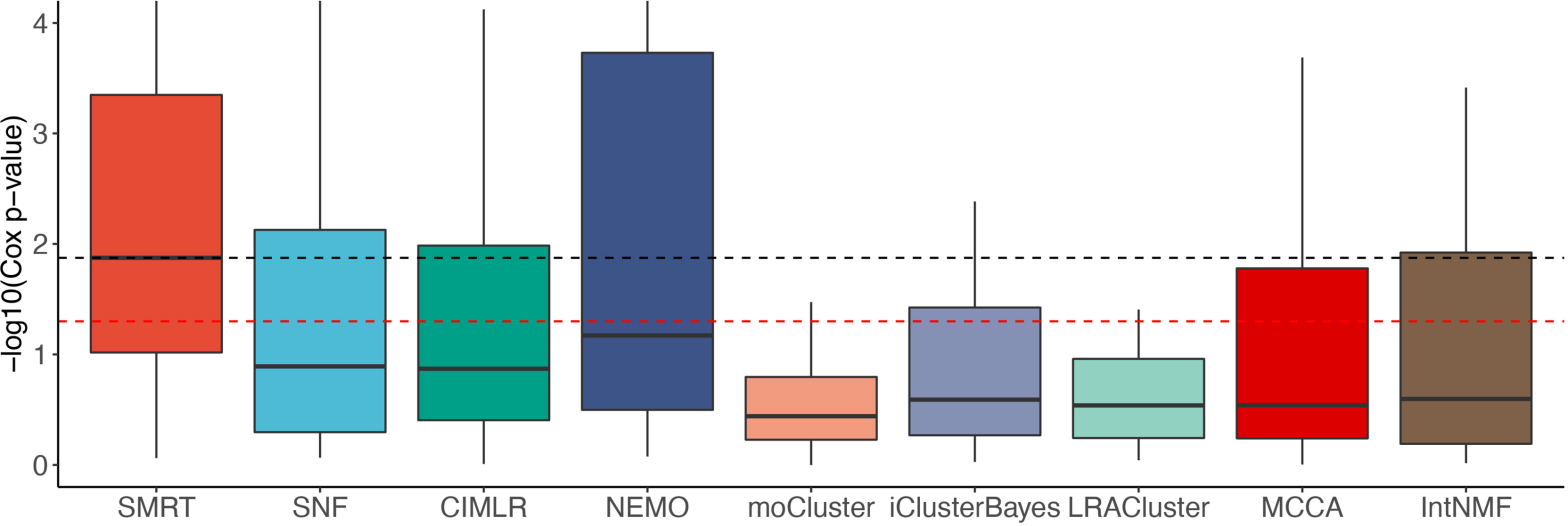
Distributions of Cox p-values (in –*log*10 scale, higher is better) of the subtypes discovered from 37 TCGA and 2 METABRIC datasets. The red dashed line shows the 5% significance level. Note that all existing methods do not reach this level of significance on average (median). Overall, the Cox p-values obtained from SMRT are substantially more significant than those of other methods (*p* = 0.0002 using the one-tailed Wilcoxon test).

**Figure 2** shows the distributions of the Cox p-values in the -log10 scale. Overall, the median -log10 p-values of SMRT is close to 2 (i.e., median p-value of 0.01) whereas the median -log10 p-value of the second-best method (NEMO) is close to 1 (i.e., median p-value of 0.1). A Wilcoxon test also confirms that the p-values of SMRT are significantly smaller than the p-values obtained from other methods (*p* = 0.0002 using the one-tailed Wilcoxon test).

The running time of each method is shown in **Table 2**. The top 39 row shows the running time of each method in each dataset while the last row shows the average running time. On average, SMRT, SNF, NEMO, and MCCA are fast and able to finish each analysis in less than a minute. The remaining methods are slower, especially iClusterBayes and IntNMF, although their analysis is limited to only 2,000 most varied genes.

**Table 2:** Running time (in minutes) of SNF, CIMLR, NEMO, moCluster, iClusterBayes (iCB), LRACluster (LRA), MCCA, IntNMF, and SMRT for 37 TCGA and two METABRIC datasets.

Dataset	Size	SNF	CIMLR	NEMO	moCluster	iCB	LRA	MCCA	IntNMF	SMRT
1. ACC	79	0.40	1.14	0.05	0.97	9.09	5.58	0.50	6.64	0.25
2. BLCA	404	0.73	3.71	0.28	7.85	29.57	34.92	0.83	21.94	1.30
3. BRCA	622	1.61	9.44	0.75	24.09	56.39	102.13	1.61	40.07	1.53
4. CESC	304	1.01	3.23	0.28	8.78	30.49	50.41	1.20	20.66	0.90
5. CHOL	36	0.33	0.60	0.02	0.38	5.23	2.02	0.53	4.77	0.10
6. COAD	220	0.93	1.84	0.20	5.28	23.77	30.81	1.07	16.44	0.67
7. COADREAD	294	0.98	4.41	0.30	9.14	29.81	40.10	1.17	21.07	0.96
8. DLBC	47	0.37	0.61	0.03	0.52	6.25	2.66	0.44	4.90	0.16
9. ESCA	183	0.75	2.44	0.14	4.45	16.91	27.54	0.84	12.93	1.20
10. GBM	273	0.05	2.15	0.02	0.46	20.30	1.02	0.19	15.03	0.91
11. GBMLGG	510	0.89	5.33	0.40	11.61	44.30	41.47	0.97	31.08	1.43
12. HNSC	228	0.84	2.24	0.18	5.41	16.32	32.22	1.06	13.51	0.77
13. KICH	65	0.37	1.13	0.03	0.70	5.93	3.47	0.47	4.93	0.33
14. KIPAN	654	1.14	13.77	0.49	14.90	41.54	63.67	1.16	31.39	3.51
15. KIRC	124	0.04	1.14	0.01	0.15	8.53	0.65	0.09	7.76	0.16
16. KIRP	271	0.61	3.93	0.15	3.96	16.85	18.91	0.70	15.96	0.94
17. LAML	164	0.04	1.57	0.01	0.20	10.84	0.68	0.10	8.13	0.13
18. LGG	510	1.29	7.60	0.60	13.95	33.18	83.92	1.37	28.77	1.76
19. LIHC	366	0.80	3.81	0.28	6.54	23.33	34.19	0.94	20.12	0.84
20. LUAD	428	0.81	4.42	0.28	7.95	34.64	39.17	1.02	29.77	1.26
21. LUSC	110	0.04	1.15	0.00	0.11	7.83	0.46	0.09	6.40	0.12
22. MESO	86	0.42	0.85	0.03	0.88	7.67	5.40	0.60	6.98	0.26
23. OV	286	0.36	2.37	0.10	3.14	19.37	16.24	0.53	16.99	0.72
24. PAAD	178	0.46	1.96	0.08	2.23	11.72	12.25	0.67	8.86	0.98
25. PCPG	179	0.55	2.35	0.12	2.52	15.98	14.51	0.64	11.79	0.52
26. PRAD	493	1.51	6.13	0.54	12.52	33.67	79.05	1.29	32.18	1.75
27. READ	74	0.39	0.86	0.03	0.64	6.32	4.24	0.59	5.88	0.22
28. SARC	257	0.54	3.07	0.14	3.29	18.00	17.82	0.63	12.64	1.40
29. SKCM	439	0.83	6.51	0.34	7.71	27.58	35.17	0.78	23.61	1.76
30. STAD	362	0.87	5.07	0.33	5.77	24.99	34.14	0.89	18.61	1.07
31. STES	545	1.55	8.79	0.53	14.11	37.81	88.00	1.22	28.85	1.85
32. TGCT	134	0.85	1.79	0.10	2.01	10.61	18.49	0.93	7.01	0.41
33. THCA	499	1.06	5.90	0.46	8.85	33.01	53.59	0.92	25.35	1.66
34. THYM	119	0.49	0.97	0.07	1.18	8.78	9.76	0.52	7.16	0.28
35. UCEC	234	1.04	2.57	0.19	4.60	19.61	34.42	1.08	14.78	0.88
36. UCS	56	0.47	0.64	0.04	0.49	6.18	3.92	0.62	4.58	0.19
37. UVM	80	0.41	0.73	0.04	0.61	7.91	5.27	0.60	6.25	0.24
38. M_Discovery	997	0.38	17.96	0.21	7.10	60.24	16.17	0.38	49.62	2.42
39. M_Validation	983	0.37	10.14	0.19	6.85	58.11	17.95	0.40	50.87	2.28
Mean	305	0.68	3.96	0.21	5.43	22.53	27.75	0.76	17.80	0.98

**Figure 3:**
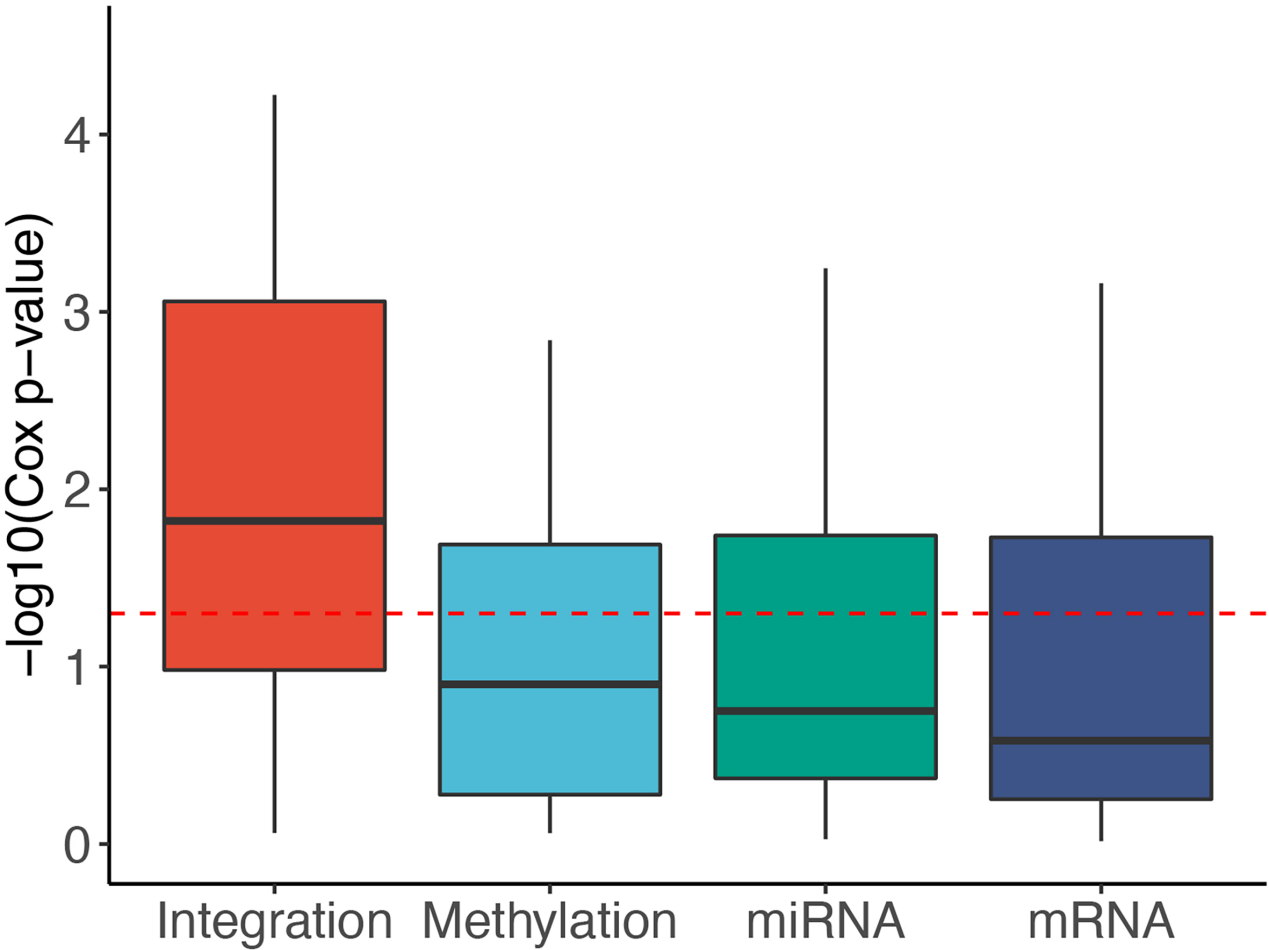
Distribution of -log10 Cox p-values for each data type of the 37 TCGA datasets. The horizontal red line indicates the significant threshold of *p*-value = 0.05. The p-values of subtypes discovered using multi-omics integration are substantially more significant than those obtained from individual data types (mRNA, methylation, miRNA).

To reveal the contribution of each data type, we used SMRT to partition the patients using each of the data types independently. Next, we calculated the Cox p-values obtained from each data type and compared them with those obtained from subtyping the multi-omics data. **Figure 3** shows the distribution of -log10 p-values of subtypes by each data type for 37 TCGA datasets. The p-values obtained from multi-omics data are substantially more significant than those obtained from individual data types. The median p-value obtained from multi-omics data is close to 0.01 (-log10 values are close to 2) while the median p-values of each data type are even higher than 0.1 (-log10 values are close to 1). This demonstrates that SMRT is able to exploit the complementary information available in each data type to determine subtypes with significant survival differences. **Supplementary Section 10** and **Table S15** provide more details on the contribution of individual data types in each dataset.

Next, we investigated the association between discovered subtypes and clinical variables. We performed our analysis on gender, age, cancer stage, and tumor grade, which are available for at least 15 datasets. We perform the following analyses: (1) Fisher’s exact test to assess the significance of the association between gender (male and female) and the discovered subtypes; (2) ANOVA to assess the age difference between discovered subtypes; and finally (3) calculate the agreement between the discovered subtypes and known cancer stages and tumor grades using Normalized Mutual Information (NMI). The distributions of –*log*10 of p-values for gender and age are shown in **Supplementary Figure S8** (see **Supplementary Tables 11-12** for the exact p-values). With the exception of NEMO and iClusterBayes, the clustering methods do not generally yield differences in gender or age in their clustering. For gender, iClusterBayes has significant p-values in 17 out of 31 datasets. For age, NEMO and iClusterBayes have significant p-values in 17 and 15 out of 29 datasets, respectively. This result demonstrates that there are meaningful and survival-related molecular signatures inside the data to be discovered, and the methods do not simply separate patients based on some visible clinical variables such as gender or age. **Supplementary Figure S9** and **Supplementary Tables 13**, **14** show the NMI values that represent the agreement between the discovered subtypes and known cancer stages and tumor grades. For the cancer stage, the median NMI values of SMRT and NEMO are comparable and are higher than the rest. For tumor grade, SMRT has the highest median NMI. However, for both cancer stage and tumor grade, the NMI values of all methods are low, meaning that there is a low agreement between the known stages/grades and the discovered subtypes using any of the subtyping methods. In conclusion, the discovered subtypes from SMRT and other subtyping methods have little agreement with clinical variables like gender, age, cancer stage, and tumor grade.

### 3.2 Case Study of the GBMLGG Dataset

**Figure 4:**
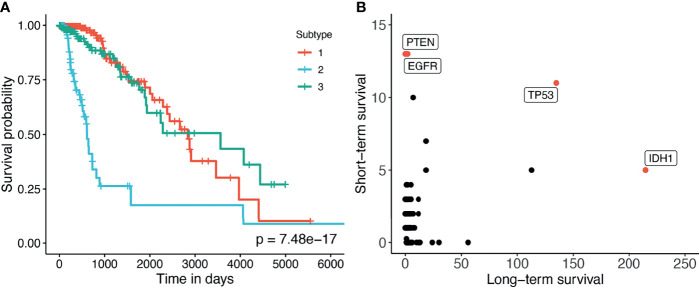
**(A)** Kaplan–Meier survival analysis of the GBMLGG dataset. The horizontal axis represents the time (days) while the vertical axis represents the estimated survival probability. **(B)** Number of patients in each group for each mutated gene in GBMLGG dataset. The horizontal axis shows the count for other subtypes with high survival rates, and the vertical axis represents the count in the subtype with low survival rates.

Here we perform an in-depth analysis for the GBMLGG (Glioma). **Figure 4A** shows the Kaplan–Meier survival analysis of the discovered subtypes. For this dataset, SMRT discovers three subtypes in which one subtype (group 2) has a very low survival rate where at year 3, the survival probability of patients this group is only at 26% while that number for the patients in the other two subtypes (groups 1 and 3) is 84%. We also perform a variant analysis for the dataset in order to find mutations that highly occur in the short-term-survival patient group (group 2) but not in the long-term-survival patient group (groups 1 and 3) and vice versa. **Figure 4B** shows the mutations of each group in which each point is a gene, and its coordinates represent the number of patients that have that mutation in the corresponding group. In principle, we want to investigate the mutated genes in the top left or bottom right of the figure. In this figure, we can easily identify four marker genes that associate with GBMLGG disease: IDH1, TP53, PTEN, and EGFR. Among those, IDH mutant (bottom-right) is known as a factor driving Low Grade Glioma (LGG) and has been used in the WHO classification system (40) to classify IDH-mutant and IDH-wildtype, which has worse prognoses. On the other hand, EGFR is not a common mutation in LGG but in GBM (Glioblastoma) (41) which has a very low survival rate (42). The amplification of EGFR can cause the mutation of PTEN gene (43) which is a tumor suppressor gene (44). Interestingly, no patient in the long-term-survival group has PTEN mutation. The occurrence of EGFR mutated genes may be another cause that leads to a low survival rate of patients in the short-term-survival group.

We further conduct pathway analysis using the discovered subtypes on the Consensus Pathway Analysis platform (45) using the FGSEA method (46) and KEGG pathway database. **Supplementary Figure S4** shows the pathways that are significant with a significance threshold of 0.5%. In this connected network, each node is a pathway and there is an edge between two pathways if they have common genes. As shown in the figure, the Glioma pathway is significantly impacted. Other pathways that have common components with the Glioma pathway, including MAPK signaling pathway, ErbB signaling pathway, Calcium signaling pathway, and Pathway in cancer, are also significantly impacted. This confirms that the subtypes discovered by SMRT have significant differences in the activity of Glioma- and cancer-related pathways. **Supplementary Section 2** and **Figures S1**–**S4** provide a more detailed analysis of this dataset.

### 3.3 Scalability of the Subtyping Methods

In order to assess the scalability of the nine subtyping methods, we generate a number of simulated datasets with a fixed number of genes/features of 5,000 and varying numbers of samples (from 1,000 to 100,000). In each dataset generated, there are three classes of samples – each with a different set of up-regulated genes. The true class information was used *a posteriori* to assess the accuracy of each clustering method. The memory of our server is limited to 376 GB.

**Figure 5:**
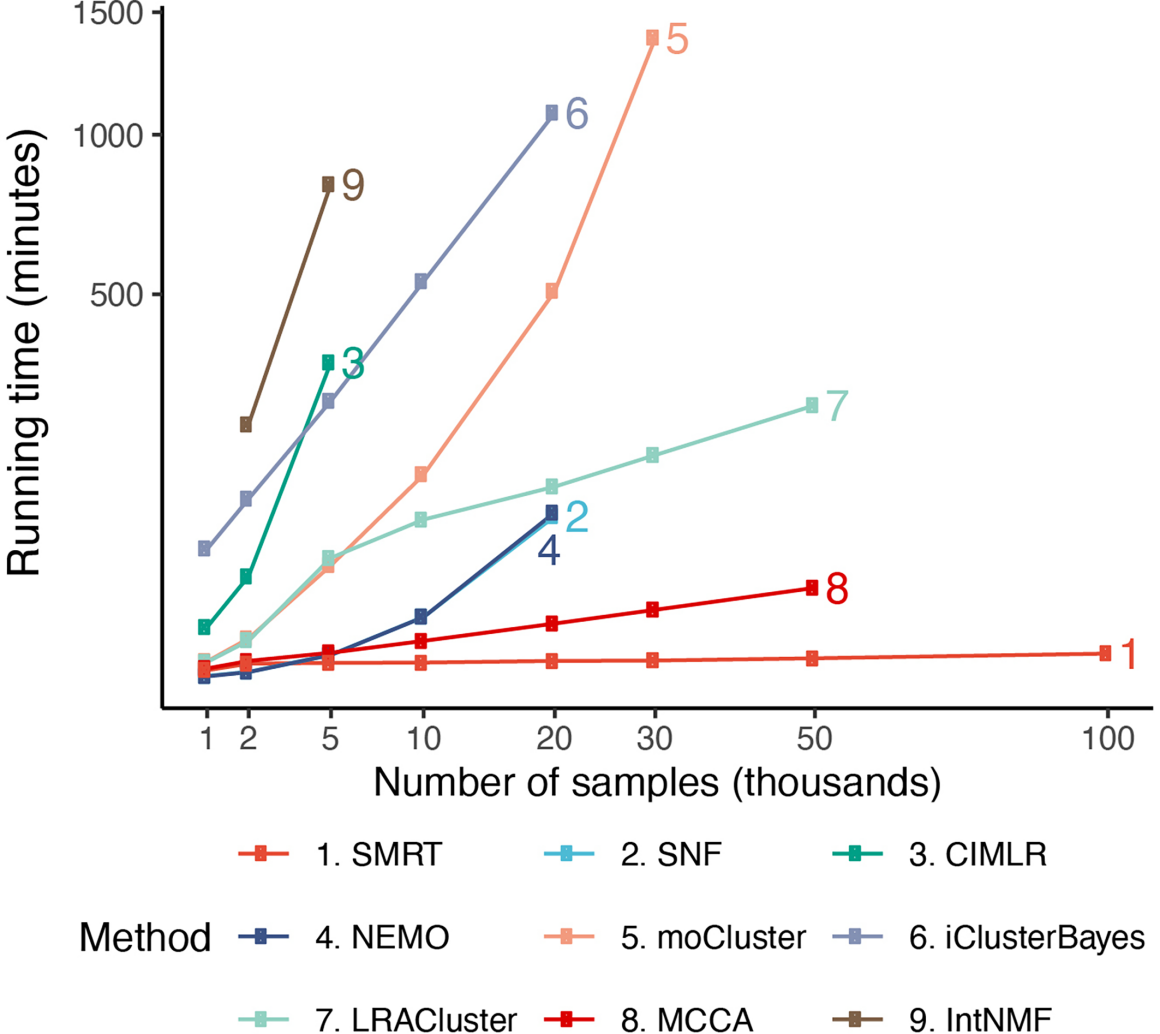
Running time of the nine subtyping methods with respect to varying numbers of samples and features. SMRT is the only method that can analyze all datasets. Even for large datasets with 100,000 samples, SMRT needs only a couple of minutes to finish the analysis.

**Figure 5** shows the running time of the methods with varying numbers of samples. The time complexity of SNF, CIMLR, NEMO, and moCluster increases exponentially with respect to sample size. These methods are not able to analyze datasets with more than 30,000 samples (out of memory, produce errors, or take more than 24 hours to analyze a single dataset). MCCA and LRACluster are able to analyze datasets with 50,000 samples but fail to analyze larger datasets. Only SMRT is able to analyze all large datasets, including those with 100,000 samples. SMRT is much faster than other methods and can analyze datasets with 100,000 samples in three minutes. See **Supplemental Section 3**, **Figure S5**, and **Tables 4**, **5** for details on simulation and results.

## 4 Conclusion

In this article, we introduced SMRT, a fast yet accurate method for data integration and subtype discovery. In an extensive analysis using 39 cancer datasets, we showed that SMRT outperformed other state-of-the-art methods in discovering novel subtypes with significantly different survival profiles. We also demonstrated that the method could accurately partition hundreds of thousands of samples in minutes with low memory requirements. At the same time, the provided web application will be extremely useful for life scientists who lack computational background or resources. Although the software was developed for the purpose of cancer subtyping, researchers in other fields can use the web application and R package for unsupervised learning and data integration.

## Data Availability Statement

Publicly available datasets were analyzed in this study. This data can be found here: http://smrt.tinnguyen-lab.com/.

## Author Contributions

HN and TN conceived of and designed the approach. HN, DT, and BT implemented the method in R, performed the data analysis and computational experiments. MR, AC, and SDa helped with data preparation and some data analysis. HN, DT, SD, and TN wrote the manuscript. All authors reviewed and approved the manuscript.

## Funding

This work was partially supported by NIH NIGMS under grant number GM103440, and by NSF under grant numbers 2001385 and 2019609.

## Author Disclaimer

Any opinions, findings, and conclusions, or recommendations expressed in this material are those of the authors and do not necessarily reflect the views of any of the funding agencies.

## Conflict of Interest

The authors declare that the research was conducted in the absence of any commercial or financial relationships that could be construed as a potential conflict of interest.

## Publisher’s Note

All claims expressed in this article are solely those of the authors and do not necessarily represent those of their affiliated organizations, or those of the publisher, the editors and the reviewers. Any product that may be evaluated in this article, or claim that may be made by its manufacturer, is not guaranteed or endorsed by the publisher.
