# Supplementary material for: SMRT: Randomized Data Transformation for Cancer Subtyping and Big Data Analysis
Source: Front Oncol. 2021 Oct 20;11:725133. doi: 10.3389/fonc.2021.725133 (PMC8563705; doi:10.3389/fonc.2021.725133)
Supplement: Supplementary file 1 [file DataSheet_1.pdf]

Supplementary Material: Randomized data transformation for cancer subtyping and big data analysis

1 DATA AND PRE-PROCESSING

In this article, we analyze 39 cancer datasets: 37 datasets from The Cancer Genome Atlas datasets (TCGA), and two datasets from The Molecular Taxonomy of Breast Cancer International Consortium (METABRIC) (Curtis et al., 2012).

We analyze 37 different types of cancer with curated level three data, available at the TCGA website (`cancergenome.nih.gov` and `firebrowse.org`): Kidney Renal Clear Cell Carcinoma (KIRC), Glioblastoma Multiforme (GBM), Acute Myeloid Leukemia (LAML), Lung Squamous Cell Carcinoma (LUSC), Bladder Urothelial Carcinoma (BLCA), Head and Neck Squamous Cell Carcinoma (HNSC), Liver Hepatocellular Carcinoma (LIHC), Stomach Adenocarcinoma (STAD), Thymoma (THYM), Glioma (GBMLGG), Brain Lower Grade Glioma (LGG), Pancreatic Adenocarcinoma (PAAD), Skin Cutaneous Melanoma (SKCM), Colorectal Adenocarcinoma (COADREAD), Uterine Corpus Endometrial Carcinoma (UCEC), Cervical Squamous Cell Carcinoma and Endocervical Adenocarcinoma (CESC), Colon Adenocarcinoma (COAD), Breast Invasive Carcinoma (BRCA), Stomach and Esophageal Carcinoma (STES), Kidney Renal Papillary Cell Carcinoma (KIRP), Kidney Chromophobe (KICH), Uveal Melanoma (UVM), Adrenocortical Carcinoma (ACC), Sarcoma (SARC), Mesothelioma (MESO), Rectum Adenocarcinoma (READ), Uterine Carcinosarcoma (UCS), Ovarian Serous Cystadenocarcinoma (OV), Esophageal Carcinoma (ESCA), Paraganglioma (PCPG), Lung Adenocarcinoma (LUAD), Prostate Adenocarcinoma (PRAD), Thyroid Carcinoma (THCA), and Testicular Germ Cell Tumors (TGCT), Cholangiocarcinoma (CHOL), Lymphoid Neoplasm Diffuse Large B-cell Lymphoma (DLBC), Pan-kidney (KIPAN). We use mRNA expression, DNA methylation, and miRNA expression data for each of the 37 cancers. For each data type, we select the platform such that it gives the largest number of patients when intersecting with patients of other data types. In the preprocessing step, only log transformation (base 2) is used if the range of the data is larger than 100 to prevent the domination of genes with extreme expression values. Table S1 shows the details of each dataset.

We also analyze two METABRIC datasets (Curtis et al., 2012), including a discovery cohort (997 patients) and a validation cohort (983 patients). For each of these patients, matched DNA and RNA were subjected to copy number analysis and transcriptional profiling on the Affymetrix SNP 6.0 and Illumina HT 12 v3 platforms, respectively. We download the mRNA and copy number variation (CNV) data from the European Genome-Phenome Archive (`www.ebi.ac.uk/ega/`) and high quality follow up clinical data from cBioPortal (`www.cbioportal.org`). There are patients that were followed up upon for almost 30 years. The only preprocessing done is mapping CNVs to genes using the CNTools package (Zhang, 2014).

When performing disease subtyping analysis with SMRT, we suggest that users use standardized data normalization, e.g., RSEM for RNA-Seq data, to process their data. We also recommend that users use log transformation (base 2) to transform the data if the range of the data is large (e.g., > 100) to mitigate unexpected effects caused by extreme expression extreme expression values on the clustering results.

Table S1. Description of 37 datasets downloaded from The Cancer Genome Atlas (TCGA).

Dataset	#Samples	mRNA	Methylation	miRNA
ACC	79	HiSeq RNASeq v2	Methylation450	HiSeq miRNASeq
BLCA	404	HiSeq RNASeq v2	Methylation450	GASeq miRNASeq
BRCA	622	HiSeq RNASeq v2	Methylation450	HiSeq miRNASeq
CHOL	36	HiSeq RNASeq v2	Methylation450	HiSeq miRNASeq
CESC	304	HiSeq RNASeq v2	Methylation450	HiSeq miRNASeq
COAD	220	HiSeq RNASeq v2	Methylation450	HiSeq miRNASeq
COADREAD	294	HiSeq RNASeq v2	Methylation450	HiSeq miRNASeq
DBLC	47	HiSeq RNASeq v2	Methylation450	HiSeq miRNASeq
ESCA	183	HiSeq RNASeq	Methylation450	HiSeq miRNASeq
GBM	273	HT HG-U133A	Methylation27	HiSeq miRNASeq
GBMLGG	510	HiSeq RNASeq v2	Methylation450	HiSeq miRNASeq
HNSC	228	HiSeq RNASeq	Methylation450	HiSeq miRNASeq
KICH	65	HiSeq RNASeq v2	Methylation450	HiSeq miRNASeq
KIPAN	654	HiSeq RNASeq	Methylation450	HiSeq miRNASeq
KIRC	124	HiSeq RNASeq	Methylation27	GASeq miRNASeq
KIRP	271	HiSeq RNASeq v2	Methylation450	HiSeq miRNASeq
LAML	164	GASeq RNASeq	Methylation27	GASeq miRNASeq
LGG	510	HiSeq RNASeq v2	Methylation450	HiSeq miRNASeq
LIHC	366	HiSeq RNASeq v2	Methylation450	HiSeq miRNASeq
LUAD	428	HiSeq RNASeq v2	Methylation450	HiSeq miRNASeq
LUSC	110	HT HG-U133A	Methylation27	GASeq miRNASeq
MESO	86	HiSeq RNASeq v2	Methylation450	HiSeq miRNASeq
OV	286	HiSeq RNASeq v2	Methylation27	HiSeq miRNASeq
PAAD	178	HiSeq RNASeq v2	Methylation450	HiSeq miRNASeq
PCPG	179	HiSeq RNASeq v2	Methylation450	HiSeq miRNASeq
PRAD	493	HiSeq RNASeq v2	Methylation450	HiSeq miRNASeq
READ	74	HiSeq RNASeq v2	Methylation450	HiSeq miRNASeq
SARC	257	HiSeq RNASeq v2	Methylation450	HiSeq miRNASeq
SKCM	439	HiSeq RNASeq v2	Methylation450	HiSeq miRNASeq
STAD	362	HiSeq RNASeq v2	Methylation450	HiSeq miRNASeq
STES	545	HiSeq RNASeq v2	Methylation450	HiSeq miRNASeq
TGCT	134	HiSeq RNASeq v2	Methylation450	HiSeq miRNASeq
THCA	499	HiSeq RNASeq v2	Methylation450	HiSeq miRNASeq
THYM	119	HiSeq RNASeq v2	Methylation450	GASeq miRNASeq
UCEC	234	GASeq RNASeq v2	Methylation450	HiSeq miRNASeq
UCS	56	HiSeq RNASeq v2	Methylation450	HiSeq miRNASeq
UVM	80	HiSeq RNASeq v2	Methylation450	HiSeq miRNASeq

Table S2. Description of the two datasets downloaded from The Molecular Taxonomy of Breast Cancer International Consortium (METABRIC).

Dataset	#Samples	mRNA	CNV
Discovery	997	Illumina HT 12 v3	Affymetrix SNP 6.0
Validation	983	Illumina HT 12 v3	Affymetrix SNP 6.0

2 ANALYSIS OF GLIOMA (GBMLGG) DATASET

Figure S1 shows the Kaplan-Meier survival analysis (Kaplan and Meier, 1958) of the discovered subtypes using the GBMLGG dataset. SMRT discovers three subtypes, each with a very different survival probability. Subtype 2 has the lowest survival rate while Subtype 3 has the highest survival rate. At year 3, patients of Subtype 2 have the survival probability at 26% while that number for patients in Subtypes 1 and 3 is 84%. Figure S2 shows the age distribution of each subtype, in which patients in Subtype 2 (low survival) are older than patients in Subtypes 1 and 3 (high survival).

We also perform variant analysis to look for mutations that are highly abundant in the short-term survival groups but not in the long-term survival groups, as shown in Figure S3. In this figure, each point represents a gene and its coordinates are the number of patients having at least a variant in that gene in each group. In principle, we would look for mutated genes in the top left and the bottom right corners. In this figure, we can easily identify four maker genes that associate with GBMLGG disease: IDH, TP53, PTEN, and EGFR. Among those, IDH mutant (bottom-right) is known as a factor driving Low Grade Glioma (LGG) and was used in the WHO classification system (Louis et al., 2016) to classify IDH-mutant and IDH-wildtype, which has worse prognoses. On the other hand, EGFR is not a common mutation in LGG but in GBM (Glioblastoma) (Hao and Guo, 2019) which has a very low survival rate (Stupp et al., 2009). The amplification of EGFR can cause the mutation of PTEN gene (Ohgaki and Kleihues, 2007) which is a tumor suppressor gene (Ali et al., 1999). Interestingly, no patient in the long-term survival group has PTEN mutation. The occurrence of EGFR mutated genes may be the cause that leads to a very low survival profile in the second group.

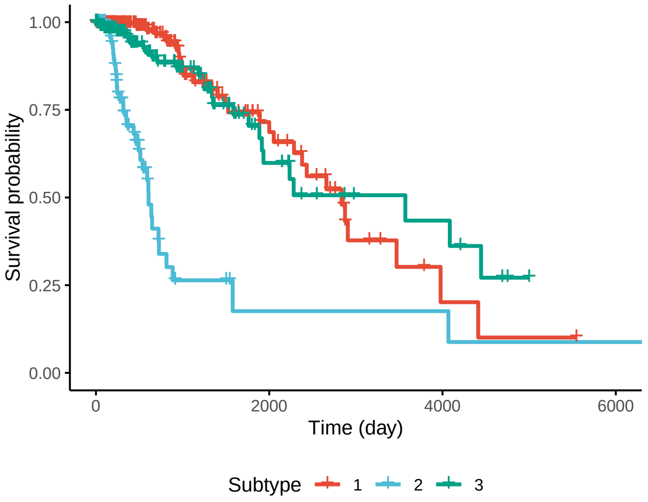

Figure S1. Kaplan-Meier survival analysis of the Glioma (GBMLGG) dataset. The horizontal axis represents the time (day) while the vertical axis represents the estimated survival probability.

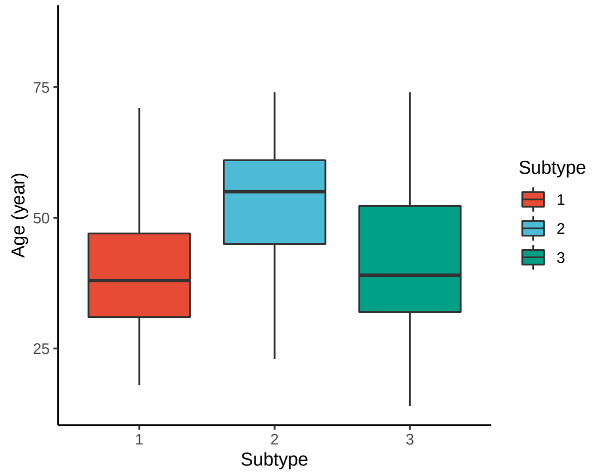

Figure S2. Age distribution for each subtype of the GBMLGG dataset.

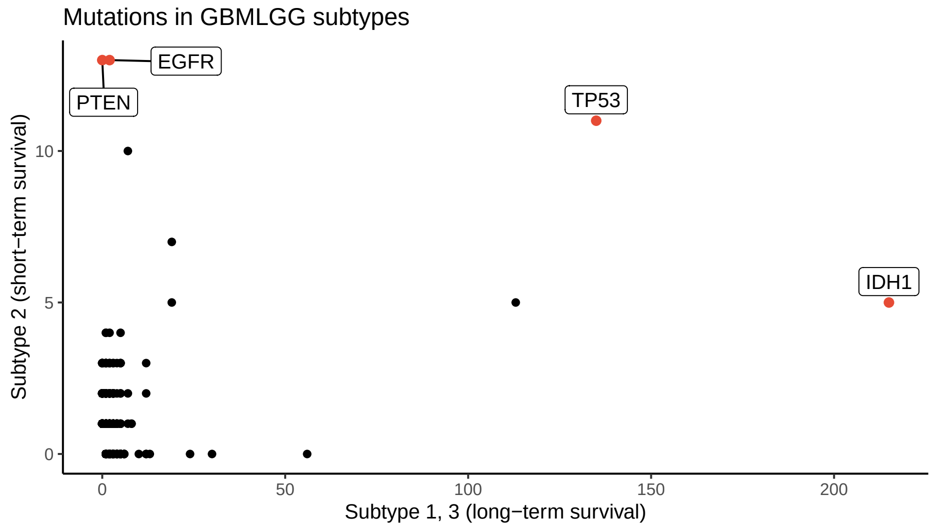

Figure S3. Number of patients in each group for each mutated gene for GBMLGG. The vertical axis represents the count in subtypes with low survival rate (subtype 2), while the horizontal axis shows the count for subtypes with high survival (subtype 1 and 3) rate.

We further investigated the contribution of genes and data types using this dataset as a case study. For this dataset, SMRT identified three subtypes using multi-omics data with a p-value of 7.48e-17. First, we map the features in miRNA data and Methylation data onto genes. For miRNA data, the mapping is done using the miRTarBase database. For methylation data, we map the methylation probes to their corresponding gene using probe positions reference genome. Next, for each of the three data types, we use an ANOVA test to calculate the signficance of each gene. This results in 3 p-values for each gene. We then combine these p-values using the Fisher's method, adjust them for multiple comparisons using false discovery rate (FDR), and ranked them according to their p-values.

Next, we performed a gene set analysis using the whole ranked list of gene and KEGG pathways. For this purpose, we used FGSEA method Korotkevich et al. (2021) implemented in our web-based platform named Consensus Pathway Analysis (CPA) Nguyen et al. (2021). Figure S4 shows the pathways that are significant with a significance threshold of 0.5%. In this connected network, each node is a pathway and there is an

edge between two pathways if they have common genes. As shown in the figure, the Glioma pathway is significantly impacted. Other pathways that have common components with the Glioma pathway, including MAPK signaling pathway, ErbB signaling pathway, Calcium signaling pathway, and Pathway in cancer, are also significantly impacted. This confirms that the subtypes discovered by SMRT have significant differences in the activity of Glioma- and cancer-related pathways. In other words, genes that belong to these pathways significantly contribute to differentiating the three subtypes. In fact, when we intersected the list of significantly differentially expressed genes from the pathways above, 5 out of 9 of the intersected list are oncogenes, including EGFR, PDGFA, PDGFB, PDGFRA, and PDGFRB Szerlip et al. (2012); Cantanhede and de Oliveira (2017); Xu and Li (2016); Carrasco-García et al. (2014); Cenciarelli et al. (2014); Verhaak et al. (2010); Yeo et al. (2021) (see Table S3 for the p-value and description of each gene).

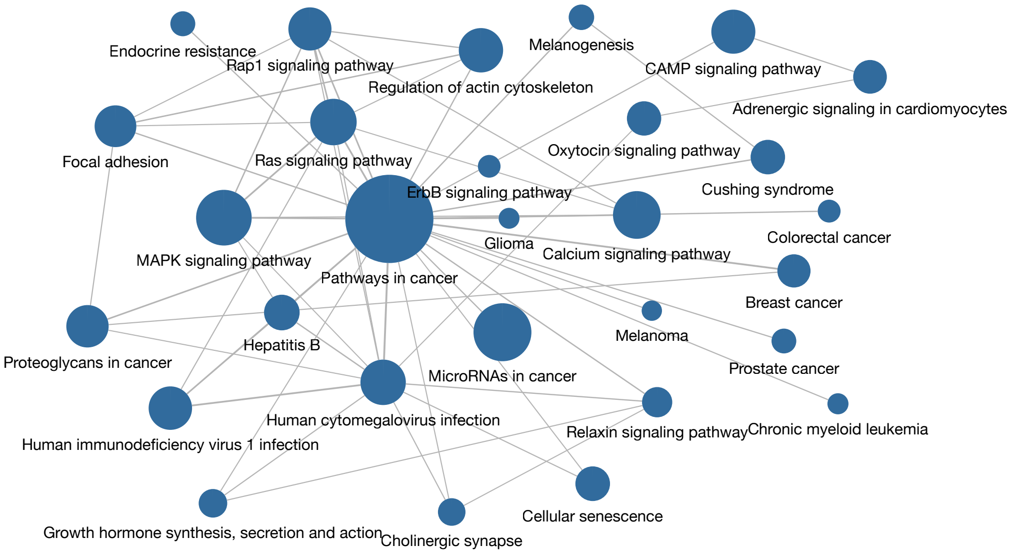

Figure S4. The largest connected component of the significant impacted pathways network resulted from pathway analysis on the subtypes discovered by SMRT using GBMLGG dataset.

Table S3. The common signficantly differential expressed genes and their p-value from the Glioma pathway, MAPK signaling pathway, Calcium signaling pathway, and Pathway in cancer.

Gene	Description	p-value.FDR
EGF	Epidermal growth factor	7.29e-43
EGFR	Epidermal growth factor receptor	2.95e-37
PDGFA	Platelet derived growth factor subunit A	1.17e-46
PDGFB	Platelet derived growth factor subunit B	6.85e-20
PDGFRA	Platelet derived growth factor receptor alpha	1.49e-49
PDGFRB	Platelet derived growth factor receptor beta	1.45e-30
PRKCA	Protein kinase C alpha	1.02e-46
PRKCB	Protein kinase C beta	4.60e-73
PRKCG	Protein kinase C gamma	2.38e-50

3 SIMULATION STUDIES

In order to test the scalability of the subtyping methods, we generate multiple datasets with varying numbers of genes and samples. The general setup is that each dataset consist of three classes (equal size), each with a different set of up-regulated genes. Figure S5 shows an example dataset of size $1,000 \times 5,000$ (1,000 samples and 5,000 genes). This dataset has three classes (sample size of 333, 333, and 334), each with a different set of 100 genes that are up-regulated. The first class has the first 100 genes up-regulated; the second class has the second 100 genes up-regulated; the third class has the third 100 genes up-regulated. The expression values of un-regulated genes follow a distribution of $\mathcal{N}(0, 1)$ ($\mu_0 = 0$, $\sigma = 1$) while the expression values of up-regulated genes follow a distribution of $\mathcal{N}(2, 1)$ ($\mu_{DE} = 2$, $\sigma = 1$).

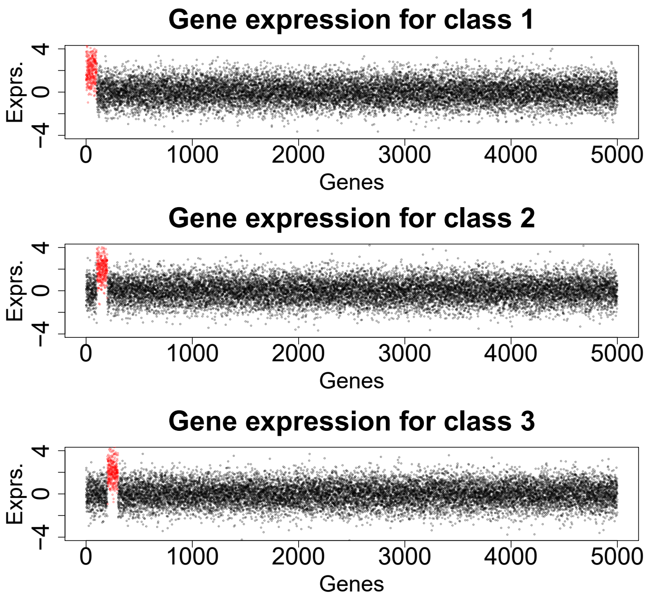

Figure S5. An example simulation. The dataset is represented as a matrix with size of $1,000 \times 5,000$ (1,000 samples and 5,000 features) divided into three classes of equal number of samples. Each class has a different set of 100 up-regulated genes (marked in red).

We test the scalability of the subtyping methods. We fix the number of genes (five thousand) but vary the number of samples (from 1,000 to 100,000). For each dataset, we use the nine subtyping methods, SNF, CIMLR, NEMO, moCluster, iClusterBayes, LRACluster, MCCA, IntNMF, and SMRT, to cluster the data. We monitor the running time and memory usage of each analysis. To assess the accuracy of the clustering methods, we compare the clustering results with the ground truth (known class label) using the Adjusted Rand Index (ARI) Hubert and Arabie (1985). The ARI takes values from -1 to 1, with the ARI expected to be 1 for a perfect agreement, and 0 for random clustering results.

The analysis results are shown in Tables S4 and S5. Table S4 shows the running time for each dataset while Table S5 shows the ARI values. IntNMF is unable to analyze dataset with more than 5,000 samples while SNF, CIMLR, NEMO, and moCluster were not able to analyze datasets with more than 30,000 samples (out of memory). Similarly, LRACluster and MCCA are unable to finish the largest dataset with 100,000 samples. Only iCB and SMRT were able to analyze all datasets. However, it takes iCB more than three days to finish the analysis of the largest dataset. SMRT is much faster than other methods. It takes

Table S4. Running time (in minutes) of the subtyping methods for simulations with 5,000 genes and varying numbers of samples (1,000 to 100,000). SNF, CIMLR, NEMO, and moCluster were not able to analyze datasets with more than 30,000 samples (out of memory). LRACluster and MCCA are unable to finish the largest dataset with 100,000 samples. Only iCB and SMRT were able to analyze all datasets. SMRT can cluster 100,000 samples in under three minutes

#Samples	SNF	CIMLR	NEMO	moCl.	iCB	LRACl.	MCCA	IntNMF	SMRT
1000	0.05	9.35	0.04	1.12	57.44	1.05	0.42	NA	0.30
2000	0.20	36.08	0.19	5.71	110.12	5.12	1.18	219.57	0.87
5000	2.06	338.86	2.05	43.94	261.61	49.94	2.43	826.91	0.94
10000	13.23	2033.37	13.19	141.47	534.02	86.29	5.06	NA	0.98
20000	89.95	NA	94.21	507.81	1081.94	125.43	10.60	NA	1.18
30000	NA	NA	NA	1386.72	1607.05	169.87	16.42	NA	1.23
50000	NA	NA	NA	NA	2660.04	253.52	28.37	NA	1.54
100000	NA	NA	NA	NA	5121.43	NA	NA	NA	2.28

Table S5. The accuracy of the clustering results measured by ARI for simulations with 5,000 genes and varying numbers of samples (1,000 to 100,000).

#Samples	SNF	CIMLR	NEMO	moCl.	iCB	LRACl.	MCCA	IntNMF	SMRT
1000	1.00	1.00	1.00	1.0000	0.86	1.00	1.00	NA	1.00
2000	1.00	1.00	1.00	1.0000	1.00	1.00	0.57	1.00	1.00
5000	1.00	1.00	1.00	1.0000	1.00	1.00	1.00	0.29	1.00
10000	1.00	1.00	1.00	1.0000	0.00	1.00	1.00	NA	1.00
20000	1.00	NA	1.00	1.0000	0.00	1.00	0.57	NA	1.00
30000	NA	NA	NA	1.0000	1.00	1.00	1.00	NA	1.00
50000	NA	NA	NA	NA	0.00	1.00	0.57	NA	1.00
100000	NA	NA	NA	NA	0.00	NA	NA	NA	1.00

SMRT less than three minutes to analyze the biggest datasets while the running time of others increase exponentially. In addition, ARI values of SMRT are maintained at 1 for all datasets.

4 SMRT AS A WEB SERVICE

The web interface SMRT is publicly accessible at `http://smrt.tinnguyen-lab.com`. We use the web-interface to forward data and requests from users to our new SMRT approach to perform data integration and clustering. Because of the efficiency of the new algorithms of SMRT, the website is able to return the results in minutes even for datasets with hundreds of thousands of samples. Figure S6 shows the main interface of the website. The interface consists of three main modules: (i) the main menu panel and file upload located on the left side, (ii) the data preview and results visualization located in the center of the website, and (iii) the data analysis panel located on the right side.

Analysis using the web application is simple and straightforward. Users can either upload expression data in $.csv$ files or a single $.rds$ file using the upload function on the left panel. Each data type is presented as a matrix in which rows represent samples and columns represent genes/features. When the input consists of a single matrix (one data type), the web interface will invoke the function *SMRT.Single()* of SMRT to partition the data using the perturbation clustering algorithm (see Section 2.3). When the input consists of multiple matrices (multi-omics data), the web application will execute the function *SMRT.Multi()* to integrate the data and determine the subtypes (see Section 2.4). SMRT can automatically determine the number of subtypes. It does not require any extra configuration or parameters to perform the analysis.

When users upload the data, the web interface will show a preview of the expression matrix and data landscape (in the first two principal components). We have embedded two built-in datasets as examples for single data type analysis (AML2004) and multiple data type analysis (KIRC). Figure S6 shows the interface of the website when the KIRC dataset is selected. This dataset has three data types: mRNA, miRNA, and DNA methylation. Users can switch to view the landscape of each data type by choosing the corresponding tab in the *Data Visualization* window.

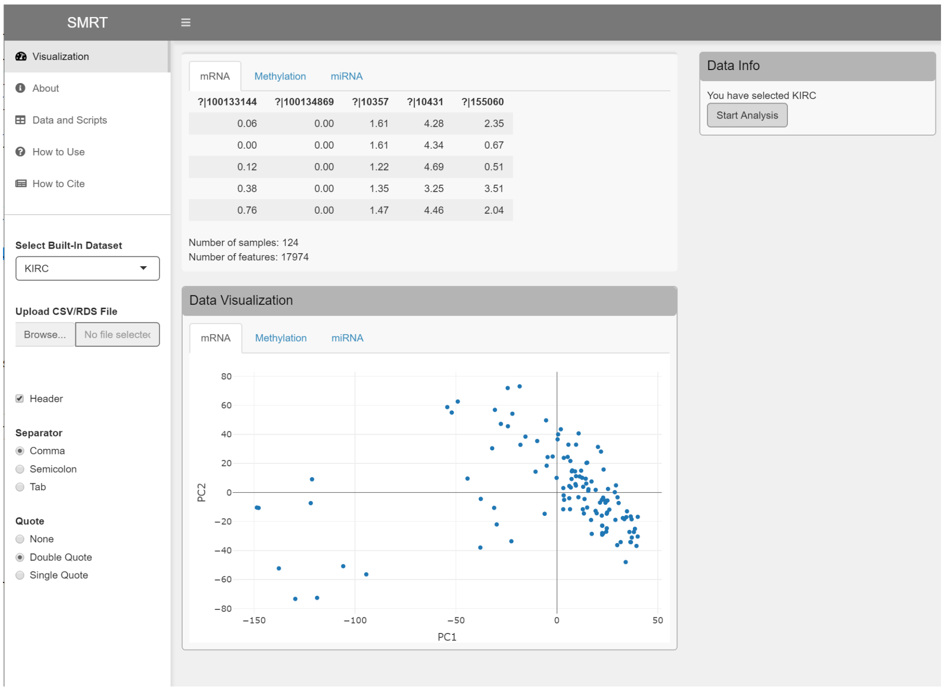

Figure S6. An overview of the SMRT web interface. The interface consists of three main modules: (i) the main menu panel located on the left side, (ii) the data viewer and related visualizations located centrally in the main body of the application, and (iii) the data analysis panel located on the right side.

When users press the *Start Analysis* button on the right panel, the web application will invoke either the *SMRT.Single()* or *SMRT.Multi()* function to partition the data. The results include subtypes and connectivity matrices for each data type. The visualization of the data landscape is updated with annotation that corresponds to the newly discovered subtypes. Figure S7 shows the analysis results from KIRC datasets with four subtypes discovered. Users can export the analysis results that include the discovered subtypes and all figures shown on the website. User can also upload clinical data or other annotation information to visualize along with the clustering results. The web application does not store any data uploaded by users. Once users close the analysis session, all related data to this session is erased from the server.

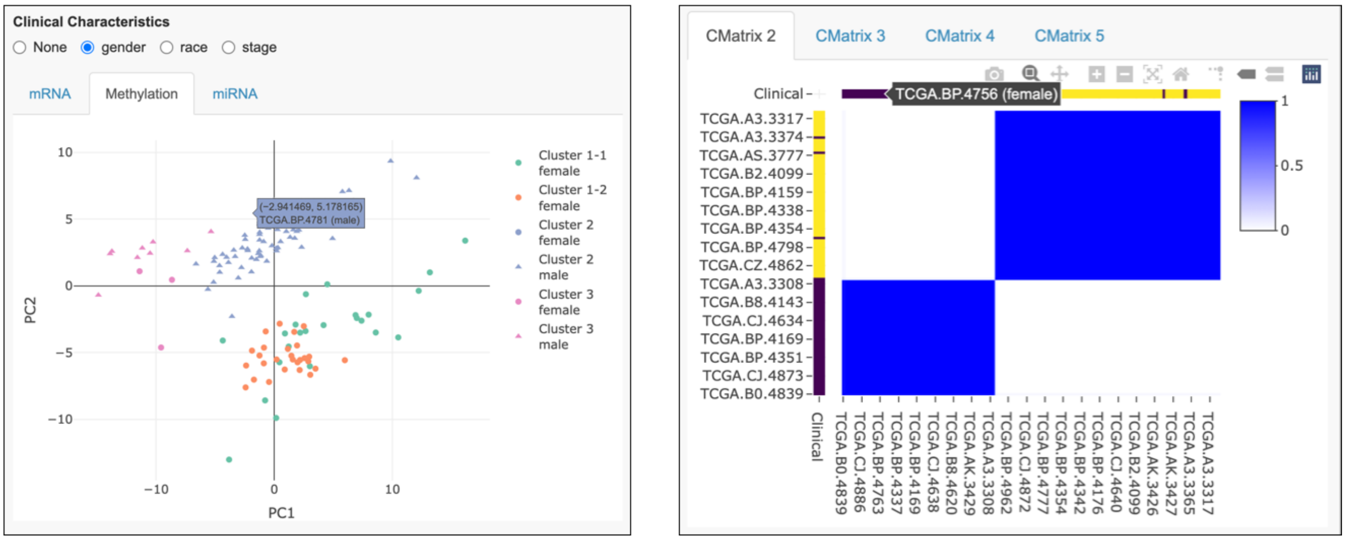

Figure S7. An overview of the subtyping result discovered by SMRT for the KIRC dataset. The left window shows the visualization of the discovered subtype with colored annotation and different exported options format. Clinical variables are shown as different shapes. The right window shows the patient connectivity matrices with the number of cluster values from 2 to 5.Left: Sample scatter plot. Clinical variables are shown in separated top and left bars.

In summary, SMRT is fast and memory efficient. This allows us to host the SMRT web interface without requiring users to have access to R runtime environment or high computational power. The web interface is user-friendly and easy-to-use. We provide a step-by-step instruction page in the *How to Use* tab of the website. We also include all data and R scripts used for our validation in the *Data and Scripts* tab of the website. New users can easily use the web application to perform unsupervised clustering for any kind of numerical data.

5 COMPARISON BETWEEN SMRT AND PINSPLUS

There are four major differences between the algorithms used in the two methods.

1. The first algorithmic difference between SMRT and our previously published method, PINSPlus, is the dimension reduction process. Both methods rely on dimension reduction to reduce the time and space complexity of cluster analysis. For this purpose, PINSPlus simply applies principal component analysis (PCA) on the input matrix. PINSPlus calculates all singular vectors from the input matrix and then uses these values to derive the low-rank decomposition of the original data. In contrast, SMRT uses randomized singular value decomposition (RSVD). Briefly, SMRT utilizes a probabilistic strategy with random projection and QR decomposition to transform an original high-dimensional matrix to a

lower-dimensional one. This smaller matrix captures the essential information of the original one. On the smaller matrix, it then calculates 20 singular vectors and uses these values to derive the low-rank decomposition of the original data. The mathematical description of the newly implemented RSVD is provided in Section 2.2 and is completely different from the PCA used in our previously published approach.

2. The second algorithmic difference between the two approaches involves the data perturbation process. Both methods rely on data perturbation to assess stability of the partitionings and then determine the best number of clusters (k). PINSPlus perturbs the data, partitions the perturbed data using a range of k, and builds a connectivity matrix for each perturbation and each value of k. While perturbing the data, PINSPlus keeps all connectivity matrices in memory (hundreds or thousands of matrices if we have tens to hundreds of iterations and 10 different values of k). In each iteration, it checks for convergence and assesses whether it is worth to perturb the data anymore. Once the data perturbation process stops, PINSPlus compares the connectivity matrices to determine with value of k (number of clusters) has the highest stability. In contrast, SMRT calculates the area under the curve (AUC) at the end of each iteration and discards all the temporary connectivity matrices. In detail, at an iteration n of each k, SMRT calculates the AUC for the perturbed connectivity matrix of this iteration and then uses that value to recalculate the average AUC from all $1...n$ iterations. Here, it only needs to store the average AUC value after each run in a small numeric vector to check for convergence. After the perturbation ends, SMRT uses the stored AUC values to determine the optimal number of k. These crucial algorithmic differences make SMRT extremely memory-efficient with respect to PINSPlus.
3. The third algorithmic difference is that SMRT has a sampling and propagating module that is completely missing in PINSPlus. This procedure is implemented for analyzing a single data type as well as multi-omics data. When the dataset is large ($> 2,000$ samples by default), SMRT splits the input into two sets: a sampled set and a propagated set. SMRT applies the dimension deduction and perturbation on the sampled set. SMRT then calculates the low-dimensional presentation of the propagated set using the rotational matrix from the sampled set. This further speeds up the dimension reduction process since SMRT does not need to apply the PCA or RSVD procedure on the whole input. When analyzing a single data type, SMRT uses a KD Tree (k-dimensional tree) to perform KNN classification to classify the propagated set using the clustering results from the sampled set. By using the KD Tree implementation, the classification process is much faster than using the naive KNN, especially when the number of samples in the propagated set increases. When analyzing multi-omics data, we select the same set of patients in all data types as the sampled set. We then perform subtyping on the sampled set and use KNN to predict the subtype of the propagated set using all data types. The subtypes of the propagated set are chosen by voting procedure (i.e., chooses subtypes with the most predictions).
4. The fourth algorithmic difference is the new module that allows SMRT to analyze data with missing values. Given a multi-omics dataset, PINSPlus requires all samples to have all data types. If some samples do not have all three data types, PINSPlus will return an error. In contrast, SMRT can handle missing values without compromising the accuracy of the method by applying a pipeline similar to the big data analysis pipeline. In detail, to cluster data that contains both match and unmatch samples, SMRT divides the input into two sets: a matched set and an unmatched set. SMRT first clusters the sampled set and then uses the KNN model described above to assign subtypes for the patients in the unmatched set. This part of the SMRT processing is completely missing from PINSPlus.

The two completely different algorithms used by SMRT and PINSPlus lead to significant differences in capabilities: (1) scalability in terms of both time and space complexity, and (2) ability to analyze unmatched

data to increase sample size and accuracy. More importantly, only SMRT can be embedded in a web-based platform due to its efficiency.

6 PERFORMANCE OF KNN WITH FIXED K AND USING ELBOW METHOD

We have performed analyses using both simulated data and real data to reflect the difference in accuracy, memory usage and running time of the two implementations (using fixed k versus using Elbow method to determine k). Table S6shows the memory usage and running time using simulated datasets. In this simulation, we generate 12 datasets with a fixed number of genes (5,000) and varying numbers of samples from 3,000 to 100,000. We set the dataset size to trigger the sampling process is 2,000 (e.g., if the dataset size is 3,000 then the size of the sampled set is 2,000 and the size of the propagated set is 1,000). It is clear that there is no difference in memory usage between the two cases. It is expected that using the Elbow method to determine k is slower than using a predefined k. However, the difference is marginal. We note that in this simulation, all ARI values are 1.

Table S7 in shows the memory usage and running time using 27 TCGA datasets. In these datasets, KNN is used to classify patients that do not have data for all data types. Again, the differences in memory usage and running time between the two cases are marginal. Regarding the accuracy, the two implementations have the sample Cox p-values in 13 datasets. Among the remaining 14, Cox p-values of $k = 10$ are better in 4 datasets and Cox p-values of k determined by the Elbow method are better in 10 datasets. Overall, using k determined by the Elbow method gives a better accuracy so we thank the Reviewer for this suggestion which brought a real improvement to our method.

Table S6. Memory usage and running time of SMRT on simulation, for two settings of KNN: (1) fixed number of neighbors ($knn.k = 10$) and (2) the number of neighbors is determined by the Elblow method. The two methods produce comparable results.

#Samples	Memory (GB)		Running Time (minutes)	
	knn.k = 10	Elbow	knn.k = 10	Elbow
3,000	1.88	1.88	1.21	1.39
4,000	2.10	2.10	1.24	1.46
5,000	2.33	2.33	1.22	1.48
6,000	2.13	2.13	1.21	1.49
7,000	2.42	2.42	1.22	1.50
8,000	2.49	2.49	1.24	1.51
9,000	2.68	2.68	1.27	1.53
10,000	2.39	2.39	1.28	1.54
20,000	3.36	3.36	1.38	1.67
30,000	5.15	5.15	1.58	1.81
50,000	8.72	8.72	1.91	2.24
100,000	17.67	17.67	2.47	2.92

Table S7. Cox p-values, memory usage and running time of SMRT on TCGA data, for two settings of KNN: (1) fixed number of neighbors ($knn.k = 10$) and (2) the number of neighbors is determined by the Elblow method. All of 27 datasets contains patients that have data of some but not all data types. Cells highlighted in yellow have significant Cox p-values at the threshold of 5%. Cells highlighted in green have the most significant Cox p-value in their respective rows. The two methods produce comparable results.

Dataset	Size		Cox p-values		Memory (GB)		Running Time (minutes)	
	Match	Total	knn.k = 10	Elbow	knn.k = 10	Elbow	knn.k = 10	Elbow
1. ACC	79	80	7.74e-03	7.74e-03	2.75	2.75	0.52	0.46
2. BLCA	404	411	1.74e-02	1.74e-02	8.89	8.89	2.37	2.10
3. BRCA	622	1095	1.33e-02	1.76e-02	21.93	21.93	3.07	3.32
4. CESC	304	307	3.20e-02	3.20e-02	9.28	9.28	1.91	1.70
5. COAD	220	301	4.54e-03	1.37e-03	7.02	7.02	1.61	1.17
6. COADREAD	294	401	7.90e-03	4.85e-03	9.27	9.27	2.03	1.94
7. DLBC	47	48	4.69e-01	4.69e-01	1.90	1.90	0.45	0.37
8. ESCA	183	185	5.01e-01	5.01e-01	5.33	5.33	2.62	2.37
9. GBMLGG	510	754	0.00e-00	0.00e-00	14.04	14.04	1.76	2.25
10. HNSC	228	527	6.86e-02	4.15e-02	10.42	8.68	1.70	1.52
11. KIPAN	654	887	9.70e-14	3.06e-13	13.41	16.10	4.66	4.62
12. KIRP	271	288	1.87e-09	1.58e-09	6.49	5.95	1.64	1.66
13. LGG	510	514	3.93e-15	3.92e-15	13.83	13.83	3.01	2.93
14. LIHC	366	376	6.96e-01	7.27e-01	8.98	8.98	1.24	1.51
15. LUAD	428	500	7.71e-01	7.71e-01	9.60	9.74	2.04	2.15
16. OV	286	571	8.69e-01	5.22e-01	4.56	4.56	1.12	1.10
17. PAAD	178	184	1.79e-04	1.79e-04	4.57	4.57	1.16	1.17
18. PRAD	493	498	3.32e-01	3.32e-01	12.74	12.74	2.91	2.92
19. READ	74	100	2.45e-03	2.45e-03	5.39	5.39	0.34	0.35
20. SARC	257	261	4.11e-02	4.11e-02	6.15	6.23	1.98	1.78
21. SKCM	439	461	9.90e-02	8.92e-02	10.9	10.9	2.60	2.17
22. STAD	362	431	3.89e-03	6.44e-04	9.50	9.06	1.68	1.59
23. STES	545	616	3.77e-02	3.81e-02	16.29	16.29	3.13	3.10
24. THCA	499	502	8.76e-02	8.74e-02	11.3	11.28	2.47	2.47
25. THYM	119	123	1.11e-02	1.11e-02	6.89	7.45	0.56	0.46
26. UCEC	234	541	2.92e-04	3.28e-05	9.55	9.55	1.19	1.34
27. UCS	56	57	3.85e-01	3.85e-01	3.88	3.81	0.38	0.32

7 ANALYSIS OF SUBTYPES FROM SMRT AND PAM50 ON BREAST CANCER DATASETS

We used the PAM50 classifier implemented in *genefu* R package Gendoo et al. (2020) to classify patients using gene expression data in the three breast cancer datasets: TCGA-BRCA and the two METABRIC datasets. Tables S8, S9 and S10 show the confusion matrix between subtypes determined by PAM50 and those discovered by SMRT. Overall, both SMRT and PAM50 are able to identify patient subgroups with significantly different survival profiles in all of the three datasets. Note that the Cox p-values of SMRT are more significant than PAM50 in two out of three datasets: 1) 0.002 (SMRT) vs. 0.009 (PAM50) for TCGA-BRCA, and 2) 3.25e-10 (SMRT) vs. 8.32e-7 (PAM50) for METABRIC_Discovery. For the METABRIC_Validation dataset, PAM50 has a marginally more significant p-value (1.77e-05 vs 2.66e-05).

For the TCGA-BRCA dataset, SMRT discovers two subtypes. Most of PAM50's Normal-like and LumA samples fall into group 1 of SMRT. Most LumB are clustered into group 2. The two remaining PAM50 subtypes (Her2 and Basal) are split equally to SMRT's two clusters. Note that the subtypes discovered by SMRT have a more significant Cox p-value than PAM50's for this dataset (0.002 vs. 0.009)

For the METABRIC Discovery dataset, SMRT discovers 6 subtypes. Most samples of Normal-like, LumA, LumB, and Her2 are grouped into groups 1, 2, 3, and 4 of SMRT, respectively. Basal samples are

split equally into groups 5 and 6. For this dataset, the Cox p-value of SMRT is more significant than that of PAM50 (3.25e-10 vs. 8.32e-7).

For the METABRIC Validation dataset, SMRT discovers 4 subtypes. Most Basal and Her2 samples belong to groups 1 and 4, respectively. The remaining subtypes (Normal-like, LumA, and LumB) are almost evenly distributed across SMRT's 4 groups. For this dataset, the Cox p-value of PAM50 is slightly more significant than that of SMRT (1.77e-5 vs. 2.66e-5).

Table S8. Confusion matrix between PAM50 subtypes and level-1 subtypes discovered by SMRT for TCGA-BRCA. The Cox p-values of subtypes obtained from PAM50 and SMRT are 0.009 and 0.002, respectively.

PAM50/SMRT	1	2
Normal-like	17	4
LumA	108	27
LumB	12	100
Her2	29	36
Basal	149	140

Table S9. Confusion matrix between PAM50 subtypes and level-1 subtypes discovered by SMRT for METABRIC Discovery dataset. Cox p-values obtained from PAM50 and SMRT are $8.32e-07$ and $3.25e-10$, respectively.

PAM50/SMRT	1	2	3	4	5	6
Normal-like	11	0	2	8	0	2
LumA	38	75	1	0	1	0
LumB	59	24	14	10	6	8
Her2	14	1	9	173	98	24
Basal	21	5	37	95	129	132

Table S10. Confusion matrix between PAM50 subtypes and level-1 subtypes discovered by SMRT for METABRIC Validation dataset. Cox p-values obtained from PAM50 and SMRT are $1.77e-05$ and $2.66e-05$, respectively.

PAM50/SMRT	1	2	3	4
Normal-like	19	20	24	69
LumA	92	114	61	69
LumB	2	5	57	88
Her2	4	1	0	41
Basal	168	35	18	96

8 CLINICAL VARIABLES ENRICHMENT ANALYSIS

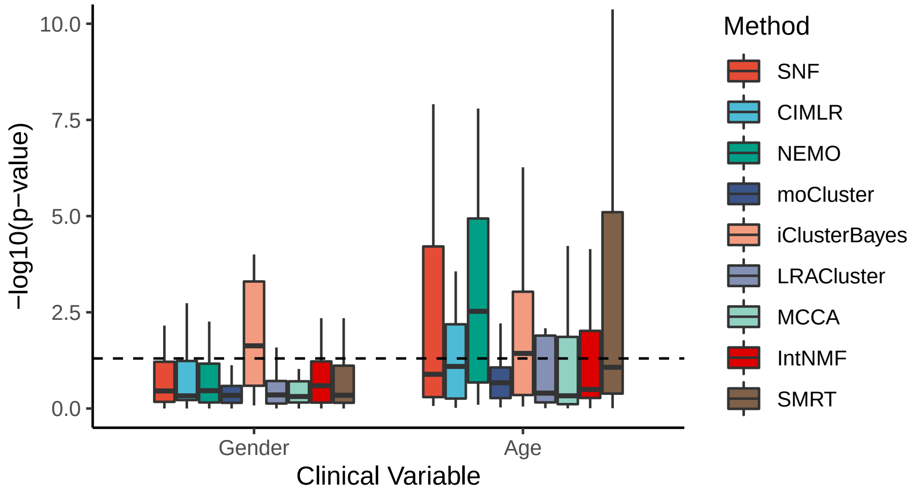

Figure S8. P-values obtained from comparing the discovered subtypes against gender, and age. Fisher's exact test was used to assess the statistical significance in the association between the discovered subtypes and gender while ANOVA was used to assess age difference. The horizontal axis shows the clinical variables while the vertical axis shows the minus $log10$ p-values. The horizontal dashed line denotes minus $log10$ of $p = 0.05$. With the exception of NEMO and iClusterBayes, the clustering methods do not generally yield differences in gender or age in their clustering.

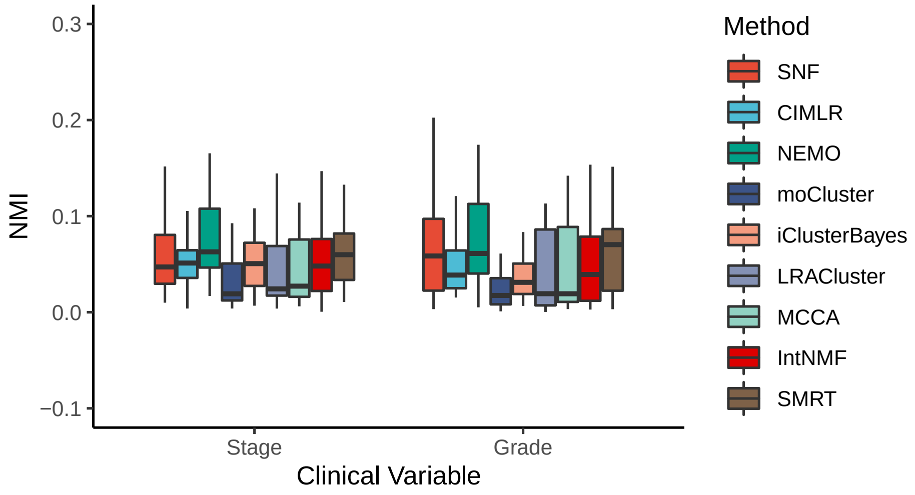

Figure S9. Normalized Mutual Information (NMI) values values obtained from comparing the discovered subtypes against known cancer stages (left panel) and tumor grades (right panel). The clustering methods do not generally yield subtypes that are correlated with cancer stages and tumor grades.

Table S11. P-values obtained from Fisher's exact test that assesses the statistical significance of the association between the discovered subtypes and gender. NA indicates that there is not enough data to perform the test or all patients have the same gender.

Dataset	SNF	CIMLR	NEMO	moCluster	iClusterBayes	LRACluster	MCCA	IntNMF	SMRT
ACC	1.1e-01	1.0e+00	2.2e-01	8.4e-01	7.7e-01	1.7e-01	6.5e-01	6.4e-02	3.8e-01
BLCA	2.6e-01	5.4e-02	2.8e-01	6.3e-01	5.0e-04	2.1e-01	1.3e-01	2.6e-01	5.0e-01
BRCA	2.1e-01	8.1e-02	2.1e-01	2.7e-02	4.9e-02	3.8e-01	2.1e-01	5.6e-02	1.0e+00
CESC	NA	NA	NA	NA	NA	NA	NA	NA	NA
CHOL	3.4e-01	1.0e+00	7.2e-01	1.0e+00	2.6e-02	7.3e-01	4.8e-01	8.9e-02	4.8e-01
COAD	7.3e-01	5.0e-01	3.3e-01	7.8e-01	5.0e-04	1.0e+00	2.7e-01	5.0e-01	6.8e-01
COADREAD	7.4e-01	9.4e-01	4.3e-01	1.7e-01	5.0e-04	5.4e-01	5.4e-01	8.1e-01	1.0e+00
DLBC	1.0e+00	3.0e-01	9.8e-01	2.5e-01	2.5e-01	5.9e-01	5.5e-01	1.0e+00	7.8e-01
ESCA	1.0e+00	8.3e-01	1.0e+00	6.5e-01	8.3e-01	1.0e+00	1.0e+00	1.0e+00	1.0e+00
GBM	7.7e-01	3.5e-01	1.0e+00	1.6e-01	1.3e-02	1.3e-01	8.1e-01	3.0e-02	5.0e-04
GBMLGG	3.6e-01	7.6e-01	3.3e-01	4.0e-01	5.0e-04	1.0e+00	5.0e-01	2.4e-01	5.4e-01
HNSC	9.6e-03	5.8e-01	3.3e-02	1.0e+00	2.8e-01	4.5e-01	9.4e-02	5.5e-01	1.8e-01
KICH	2.0e-01	1.0e+00	4.7e-01	5.1e-01	5.3e-02	1.7e-01	6.1e-01	9.4e-02	4.5e-01
KIPAN	4.5e-02	5.7e-02	2.8e-02	1.0e+00	5.0e-04	3.4e-02	3.6e-01	7.5e-03	2.5e-03
KIRC	2.7e-01	6.6e-07	1.7e-02	3.0e-01	2.7e-01	3.0e-01	1.8e-01	1.3e-01	5.0e-04
KIRP	1.1e-03	7.0e-05	5.0e-04	4.6e-01	5.0e-04	1.2e-04	1.7e-05	5.0e-04	2.3e-02
LAML	4.3e-01	4.2e-01	1.8e-01	6.2e-01	8.6e-02	1.0e+00	5.2e-01	7.5e-01	9.4e-01
LGG	3.7e-01	4.9e-01	3.7e-01	2.5e-02	5.0e-04	4.4e-01	8.9e-01	5.3e-04	3.3e-01
LIHC	2.9e-05	5.0e-04	5.0e-04	5.8e-01	1.0e-03	7.1e-01	1.6e-03	1.7e-01	2.0e-01
LUAD	1.0e-06	5.0e-04	5.0e-04	5.9e-04	5.0e-04	5.0e-01	1.0e+00	5.2e-02	3.4e-02
LUSC	9.1e-02	5.0e-04	5.2e-01	1.0e+00	1.9e-01	4.3e-01	3.7e-01	3.9e-01	7.6e-02
MESO	7.6e-01	7.7e-02	1.2e-01	1.9e-01	7.0e-01	7.6e-01	5.0e-01	3.8e-01	1.0e+00
OV	NA	NA	NA	NA	NA	NA	NA	NA	NA
PAAD	5.0e-03	1.4e-01	3.8e-01	6.8e-02	3.1e-01	7.1e-01	8.5e-49	9.5e-01	3.3e-01
PCPG	5.3e-01	7.3e-01	7.6e-01	1.0e+00	5.0e-04	2.4e-01	4.5e-01	8.8e-01	1.0e+00
PRAD	NA	NA	NA	NA	NA	NA	NA	NA	NA
READ	1.0e+00	5.6e-01	2.5e-01	3.7e-01	1.5e-03	3.0e-01	9.5e-01	1.0e+00	5.9e-01
SARC	5.0e-04	1.5e-03	5.0e-04	4.6e-01	5.0e-04	1.5e-05	1.7e-03	7.7e-04	2.5e-03
SKCM	4.1e-01	5.3e-01	7.3e-01	5.5e-01	4.7e-02	1.4e-01	4.9e-01	4.0e-01	1.5e-03
STAD	5.6e-01	6.3e-01	9.1e-01	2.7e-01	1.7e-01	3.4e-01	1.0e+00	1.9e-01	1.2e-01
STES	6.0e-04	1.8e-03	3.5e-03	4.6e-01	9.9e-05	5.6e-02	3.1e-03	5.1e-04	9.1e-02
TGCT	NA	NA	NA	NA	NA	NA	NA	NA	NA
THCA	3.7e-01	4.4e-01	7.1e-01	5.5e-01	6.0e-01	7.9e-01	7.5e-01	7.9e-02	5.7e-01
THYM	6.4e-01	5.2e-01	1.9e-01	3.3e-01	6.0e-03	1.0e+00	2.4e-01	8.5e-01	7.1e-01
UCEC	NA	NA	NA	NA	NA	NA	NA	NA	NA
UCS	NA	NA	NA	NA	NA	NA	NA	NA	NA
UVM	1.0e+00	5.4e-01	1.0e+00	1.0e+00	6.4e-01	9.7e-01	1.0e+00	6.5e-01	7.5e-01
METABRIC validation	NA	NA	NA	NA	NA	NA	NA	NA	NA
METABRIC discovery	NA	NA	NA	NA	NA	NA	NA	NA	NA
# significant	8	7	8	3	17	3	5	6	7

Table S12. P-values obtained from ANOVA test that assesses the statistical significance of the association between the discovered subtypes and age. NA indicates that there is not enough data to perform the test.

Dataset	SNF	CIMLR	NEMO	moCluster	iClusterBayes	LRACluster	MCCA	IntNMF	SMRT
ACC	NA	NA	NA	NA	NA	NA	NA	NA	NA
BLCA	6.6e-03	7.1e-03	1.8e-07	2.0e-02	2.8e-02	2.8e-02	1.4e-02	8.4e-02	5.5e-02
BRCA	2.0e-01	2.7e-04	8.6e-02	2.1e-01	2.3e-05	1.3e-01	9.1e-02	7.1e-02	6.4e-02
CESC	3.8e-01	1.3e-01	5.9e-01	8.4e-01	5.9e-09	4.0e-01	7.7e-01	4.3e-01	7.6e-01
CHOL	NA	NA	NA	NA	NA	NA	NA	NA	NA
COAD	5.4e-01	2.2e-02	2.1e-01	9.3e-02	2.2e-01	8.5e-01	4.7e-01	3.8e-01	8.1e-01
COADREAD	7.6e-01	6.1e-01	8.0e-01	8.1e-01	6.4e-01	7.2e-01	6.8e-01	5.3e-01	5.2e-01
DLBC	8.6e-01	8.3e-01	7.1e-01	5.4e-01	6.5e-01	7.4e-01	8.7e-01	5.9e-01	4.1e-01
ESCA	NA	NA	NA	NA	NA	NA	NA	NA	NA
GBM	1.4e-02	2.9e-02	1.1e-03	4.1e-01	5.2e-05	9.0e-03	9.9e-01	9.6e-03	4.2e-11
GBMLGG	1.2e-17	2.8e-16	1.7e-17	8.7e-08	5.4e-07	4.5e-09	7.6e-10	7.3e-07	1.5e-13
HNSC	5.3e-01	5.6e-02	2.6e-03	9.4e-01	7.5e-01	5.5e-01	8.3e-01	7.3e-01	6.6e-01
KICH	3.0e-01	8.1e-02	1.4e-01	1.3e-01	7.3e-01	3.3e-01	2.2e-01	4.0e-01	3.3e-01
KIPAN	1.2e-08	8.8e-08	6.5e-07	8.6e-02	5.1e-10	3.9e-08	5.1e-01	1.1e-08	5.8e-08
KIRC	6.1e-01	5.9e-01	3.1e-01	5.6e-01	8.9e-01	5.6e-01	6.2e-01	7.8e-01	1.8e-02
KIRP	2.3e-01	9.6e-01	1.2e-05	1.1e-01	2.9e-02	9.8e-01	9.5e-01	5.5e-04	2.1e-03
LAML	6.1e-05	1.1e-01	2.2e-05	6.1e-03	5.8e-02	1.3e-02	6.3e-03	5.3e-03	7.9e-06
LGG	1.9e-18	3.3e-16	2.2e-19	6.7e-01	9.2e-04	8.8e-03	5.2e-13	6.1e-01	1.9e-09
LIHC	3.3e-05	6.5e-03	3.6e-04	2.9e-01	4.6e-03	9.0e-01	6.0e-05	9.1e-01	2.1e-04
LUAD	1.5e-02	2.7e-02	6.0e-02	3.1e-02	2.2e-02	3.3e-01	9.0e-01	2.3e-02	5.8e-01
LUSC	8.0e-01	4.0e-01	5.8e-01	2.0e-01	6.8e-01	6.9e-01	6.3e-01	5.1e-01	2.7e-01
MESO	NA	NA	NA	NA	NA	NA	NA	NA	NA
OV	1.3e-01	1.3e-01	2.0e-01	6.9e-01	1.1e-05	9.2e-01	4.4e-04	2.3e-01	9.8e-01
PAAD	5.0e-01	5.5e-01	4.4e-01	2.0e-01	3.4e-01	9.1e-02	8.7e-01	4.8e-01	1.6e-01
PCPG	NA	NA	NA	NA	NA	NA	NA	NA	NA
PRAD	1.6e-01	5.7e-01	6.0e-04	4.8e-01	1.6e-02	4.6e-01	3.1e-01	4.7e-01	8.5e-02
READ	7.8e-01	8.2e-01	2.2e-01	1.7e-01	1.7e-01	4.6e-01	2.5e-02	9.8e-01	8.2e-01
SARC	NA	NA	NA	NA	NA	NA	NA	NA	NA
SKCM	6.1e-03	1.4e-01	1.0e-03	2.0e-02	3.7e-04	8.2e-03	8.5e-01	7.2e-05	1.5e-01
STAD	1.2e-04	6.0e-03	3.8e-03	2.9e-02	5.3e-01	5.0e-01	4.0e-02	1.9e-02	1.3e-01
STES	5.1e-01	8.8e-01	3.9e-02	4.6e-01	4.5e-01	4.9e-01	7.2e-01	9.6e-01	3.6e-01
TGCT	NA	NA	NA	NA	NA	NA	NA	NA	NA
THCA	9.5e-02	1.3e-02	3.0e-03	5.6e-01	2.9e-01	3.4e-01	6.4e-01	3.2e-01	4.5e-04
THYM	NA	NA	NA	NA	NA	NA	NA	NA	NA
UCEC	1.3e-07	4.1e-01	1.6e-08	2.8e-01	3.7e-02	8.7e-01	3.4e-02	7.5e-02	3.6e-06
UCS	NA	NA	NA	NA	NA	NA	NA	NA	NA
UVM	NA	NA	NA	NA	NA	NA	NA	NA	NA
METABRIC validation	1.1e-16	1.6e-15	7.0e-14	3.1e-01	4.2e-01	1.1e-14	1.6e-19	1.0e-19	2.2e-15
METABRIC discovery	1.9e-17	1.5e-03	4.7e-17	3.3e-02	2.3e-02	3.2e-14	3.3e-13	1.1e-12	6.1e-20
# significant	13	13	17	7	15	9	11	10	12

Table S13. Normalized Mutual Information (NMI) vlaues obtained from comparing the discovered subtypes against known cancer stages. NA indicates that there is not enough data to perform the calculation.

Dataset	SNF	CIMLR	NEMO	moCluster	iClusterBayes	LRACluster	MCCA	IntNMF	SMRT
ACC	0.1	0.13	0.26	0.17	0.03	0.05	0.11	0.05	0.06
BLCA	0.03	0.04	0.06	0	0.02	0.03	0.05	0.03	0.04
BRCA	0.02	0.04	0.02	0.02	0.02	0.01	0.01	0.01	0.02
CESC	0.03	0.06	0.02	0.01	0.05	0.02	0.03	0.03	0.06
CHOL	0.08	0.16	0.23	0.18	0.21	0.12	0.18	0.22	0.13
COAD	0.06	0.05	0.05	0.02	0.06	0.02	0.02	0.03	0.03
COADREAD	0.04	0.05	0.03	0.03	0.06	0.02	0.02	0.03	0.01
DLBC	0.07	0.02	0.27	0.02	0.07	0.08	0.04	0.05	0.06
ESCA	0.09	0.09	0.09	0.09	0.09	0.08	0.09	0.09	0.08
GBM	NA	NA	NA	NA	NA	NA	NA	NA	NA
GBMLGG	NA	NA	NA	NA	NA	NA	NA	NA	NA
HNSC	0.01	0.04	0.07	0.03	0.02	0.02	0.02	0.02	0.01
KICH	0.1	0.04	0.24	0.11	0.09	0.02	0.01	0.07	0.06
KIPAN	0.06	0.05	0.06	0.01	0.03	0.06	0.02	0.05	0.07
KIRC	0.04	0	0.06	0.02	0.02	0.02	0.02	0.08	0.09
KIRP	0.1	0.07	0.12	0.01	0.07	0.1	0.08	0.07	0.13
LAML	NA	NA	NA	NA	NA	NA	NA	NA	NA
LGG	NA	NA	NA	NA	NA	NA	NA	NA	NA
LIHC	0.02	0.03	0.05	0.01	0.03	0.01	0.02	0.01	0.03
LUAD	0.01	0.03	0.06	0.01	0.03	0.02	0.01	0.03	0.02
LUSC	0.04	0.05	0.06	0.03	0.04	0.06	0.04	0.05	0.07
MESO	0.02	0.06	0.12	0.01	0.06	0.02	0.09	0.08	0.12
OV	0.05	0.04	0.05	0.02	0.05	0.02	0.02	0.02	0.04
PAAD	0.08	0.06	0.1	0.09	0.1	0.1	0.02	0.09	0.09
PCPG	NA	NA	NA	NA	NA	NA	NA	NA	NA
PRAD	NA	NA	NA	NA	NA	NA	NA	NA	NA
READ	0.17	0.14	0.17	0.06	0.11	0.14	0.21	0.15	0.12
SARC	NA	NA	NA	NA	NA	NA	NA	NA	NA
SKCM	0.03	0.05	0.08	0.02	0.02	0.02	0.01	0.05	0.07
STAD	0.05	0.02	0.02	0.01	0.04	0.02	0.02	0.02	0.02
STES	0.05	0.05	0.06	0.03	0.05	0.02	0.05	0.04	0.04
TGCT	0.09	0.08	0.09	0.07	0.1	0.06	0.08	0.08	0.1
THCA	0.03	0.05	0.04	0.01	0.03	0	0.03	0	0.05
THYM	NA	NA	NA	NA	NA	NA	NA	NA	NA
UCEC	0.04	0.05	0.03	0.04	0.05	0.03	0.04	0.06	0.05
UCS	0.15	0.11	0.13	0.06	0.2	0.08	0.11	0.16	0.08
UVM	0.08	0.11	0.08	0.02	0.08	0.09	0.07	0.06	0.07
METABRIC validation	0.02	0.02	0.03	0	0.01	0.02	0.01	0.01	0.03
METABRIC discovery	0.02	0.01	0.02	0.01	0.01	0.01	0.01	0.02	0.03
Mean	0.06	0.06	0.09	0.04	0.06	0.04	0.05	0.06	0.06

Table S14. Normalized Mutual Information (NMI) vlaues obtained from comparing the discovered subtypes against known tumor grades. NA indicates that there is not enough data to perform the calculation.

Dataset	SNF	CIMLR	NEMO	moCluster	iClusterBayes	LRACluster	MCCA	IntNMF	SMRT
ACC	NA	NA	NA	NA	NA	NA	NA	NA	NA
BLCA	0.1	0.09	0.12	0.02	0.02	0.09	0.09	0.06	0.07
BRCA	NA	NA	NA	NA	NA	NA	NA	NA	NA
CESC	0.01	0.04	0.01	0.01	0.04	0	0.01	0.01	0.03
CHOL	NA	NA	NA	NA	NA	NA	NA	NA	NA
COAD	NA	NA	NA	NA	NA	NA	NA	NA	NA
COADREAD	NA	NA	NA	NA	NA	NA	NA	NA	NA
DLBC	NA	NA	NA	NA	NA	NA	NA	NA	NA
ESCA	NA	NA	NA	NA	NA	NA	NA	NA	NA
GBM	NA	NA	NA	NA	NA	NA	NA	NA	NA
GBMLGG	0.06	0.06	0.06	0.06	0.08	0.1	0.01	0.08	0.07
HNSC	0.02	0.04	0.06	0.04	0.03	0.02	0.03	0.01	0.01
KICH	NA	NA	NA	NA	NA	NA	NA	NA	NA
KIPAN	0.02	0.02	0.03	0.02	0.05	0.02	0.02	0.01	0.07
KIRC	0.1	0.02	0.13	0.11	0.08	0.11	0.11	0.11	0.11
KIRP	NA	NA	NA	NA	NA	NA	NA	NA	NA
LAML	NA	NA	NA	NA	NA	NA	NA	NA	NA
LGG	0.06	0.06	0.06	0	0.02	0	0.01	0.01	0.03
LIHC	0.01	0.03	0.03	0.01	0.05	0	0.01	0	0.02
LUAD	NA	NA	NA	NA	NA	NA	NA	NA	NA
LUSC	NA	NA	NA	NA	NA	NA	NA	NA	NA
MESO	NA	NA	NA	NA	NA	NA	NA	NA	NA
OV	0.02	0.02	0.02	0.03	0.02	0.02	0.02	0.01	0.02
PAAD	0.1	0.06	0.08	0.09	0.07	0.08	0.02	0.04	0.08
PCPG	NA	NA	NA	NA	NA	NA	NA	NA	NA
PRAD	NA	NA	NA	NA	NA	NA	NA	NA	NA
READ	NA	NA	NA	NA	NA	NA	NA	NA	NA
SARC	NA	NA	NA	NA	NA	NA	NA	NA	NA
SKCM	NA	NA	NA	NA	NA	NA	NA	NA	NA
STAD	0.05	0.04	0.05	0.01	0.03	0.01	0.02	0.05	0
STES	0	0.02	0.05	0.02	0.01	0.01	0	0.01	0.01
TGCT	NA	NA	NA	NA	NA	NA	NA	NA	NA
THCA	NA	NA	NA	NA	NA	NA	NA	NA	NA
THYM	NA	NA	NA	NA	NA	NA	NA	NA	NA
UCEC	0.2	0.05	0.17	0.01	0.03	0	0.14	0.15	0.15
UCS	NA	NA	NA	NA	NA	NA	NA	NA	NA
UVM	NA	NA	NA	NA	NA	NA	NA	NA	NA
METABRIC validation	0.1	0.12	0.12	0.01	0.01	0.06	0.09	0.08	0.1
METABRIC discovery	0.09	0.1	0.11	0.01	0.01	0.11	0.11	0.09	0.09
Mean	0.06	0.05	0.07	0.03	0.04	0.04	0.05	0.05	0.06

9 SURVIVAL ANALYSIS

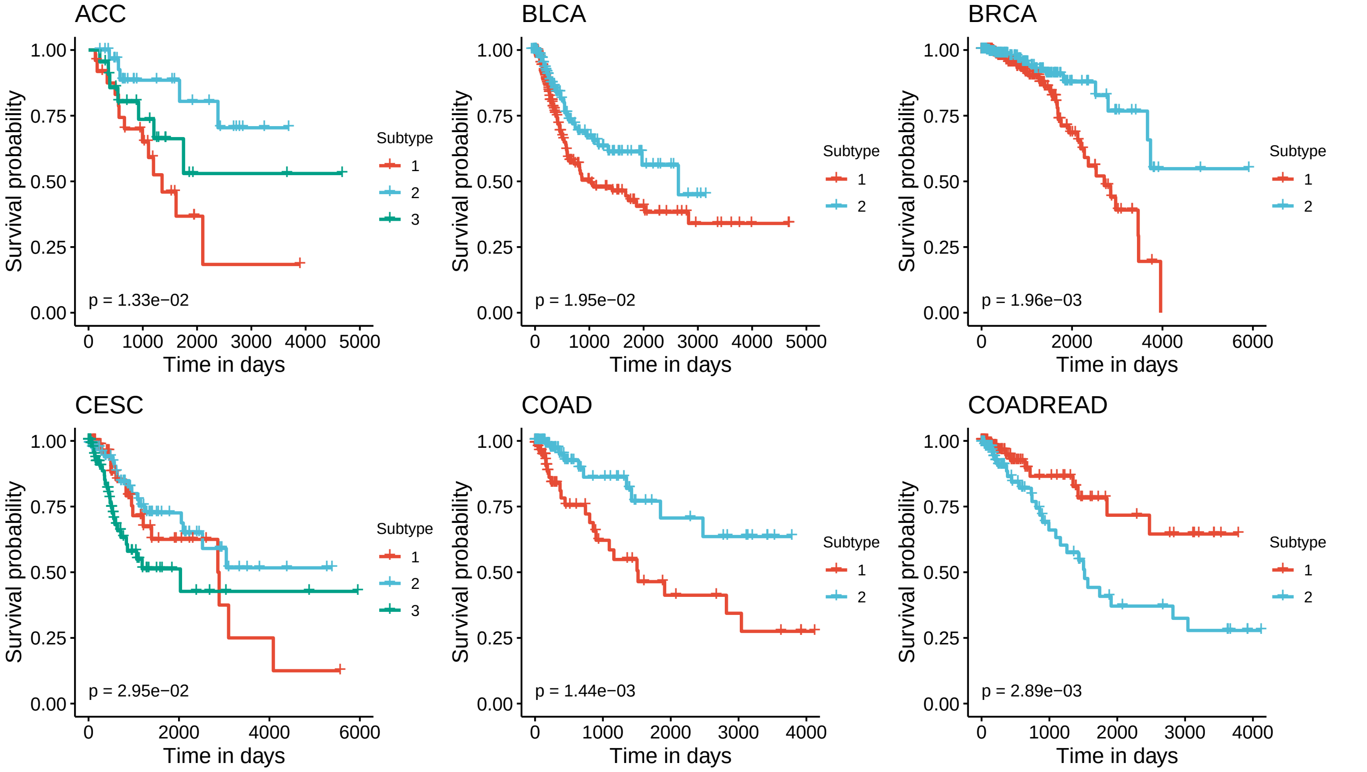

Figure S10. Kaplan-Meier survival analysis for TCGA-ACC, BLCA, BRCA, CESC, and COAD datasets.

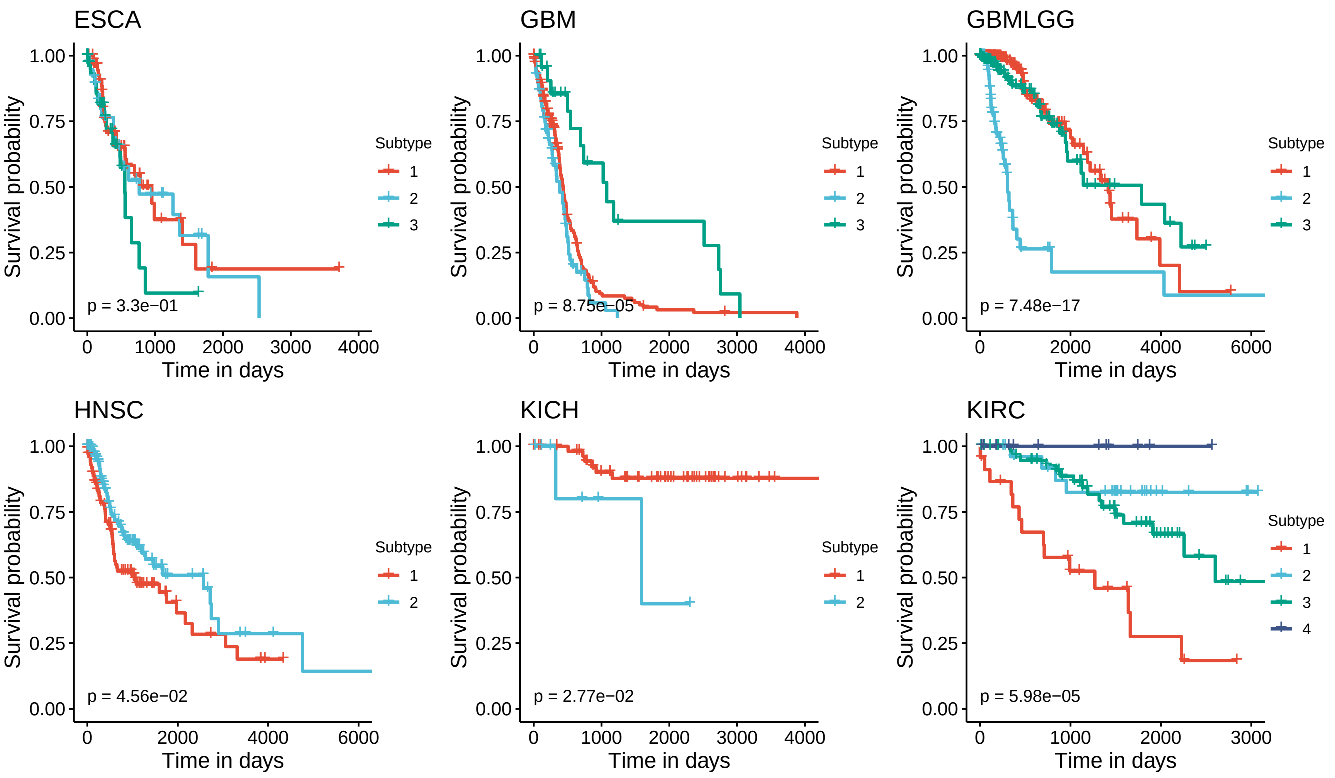

Figure S11. Kaplan-Meier survival analysis for TCGA-ESCA, GBM, GBMLGG, HNSC, KICH, and KIRC datasets.

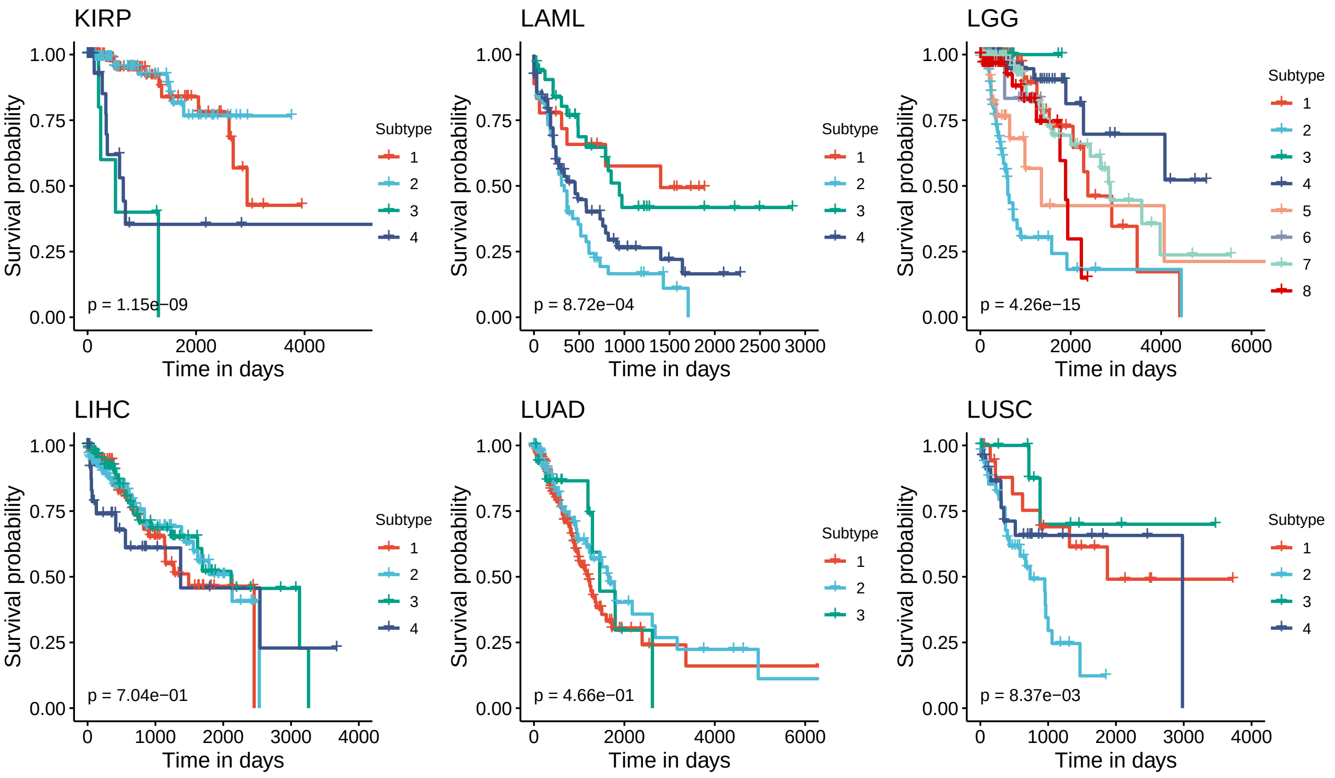

Figure S12. Kaplan-Meier survival analysis for TCGA-KIRP, LAML, LGG, LIHC, LUAD, and LUSC datasets.

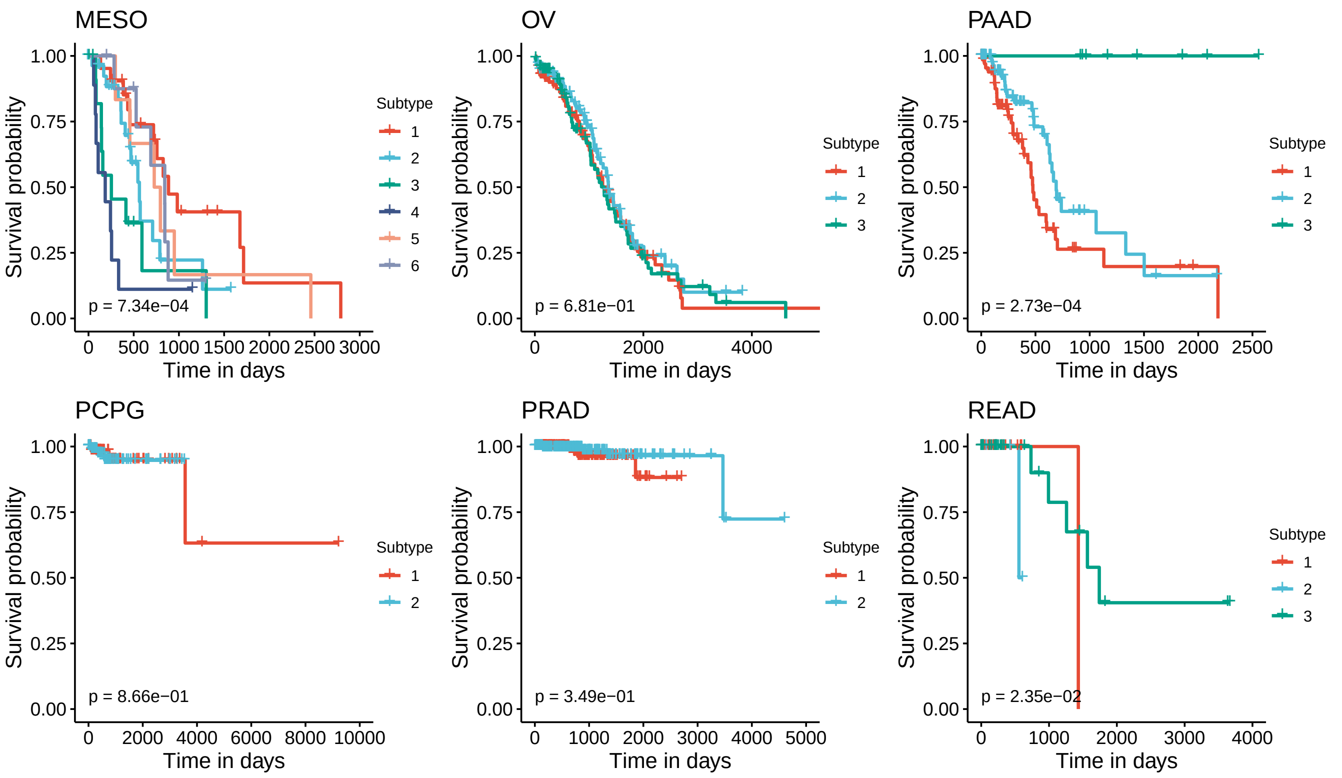

Figure S13. Kaplan-Meier survival analysis for TCGA-MESO, OV, PADD, PCPG, PRAD, and READ datasets.

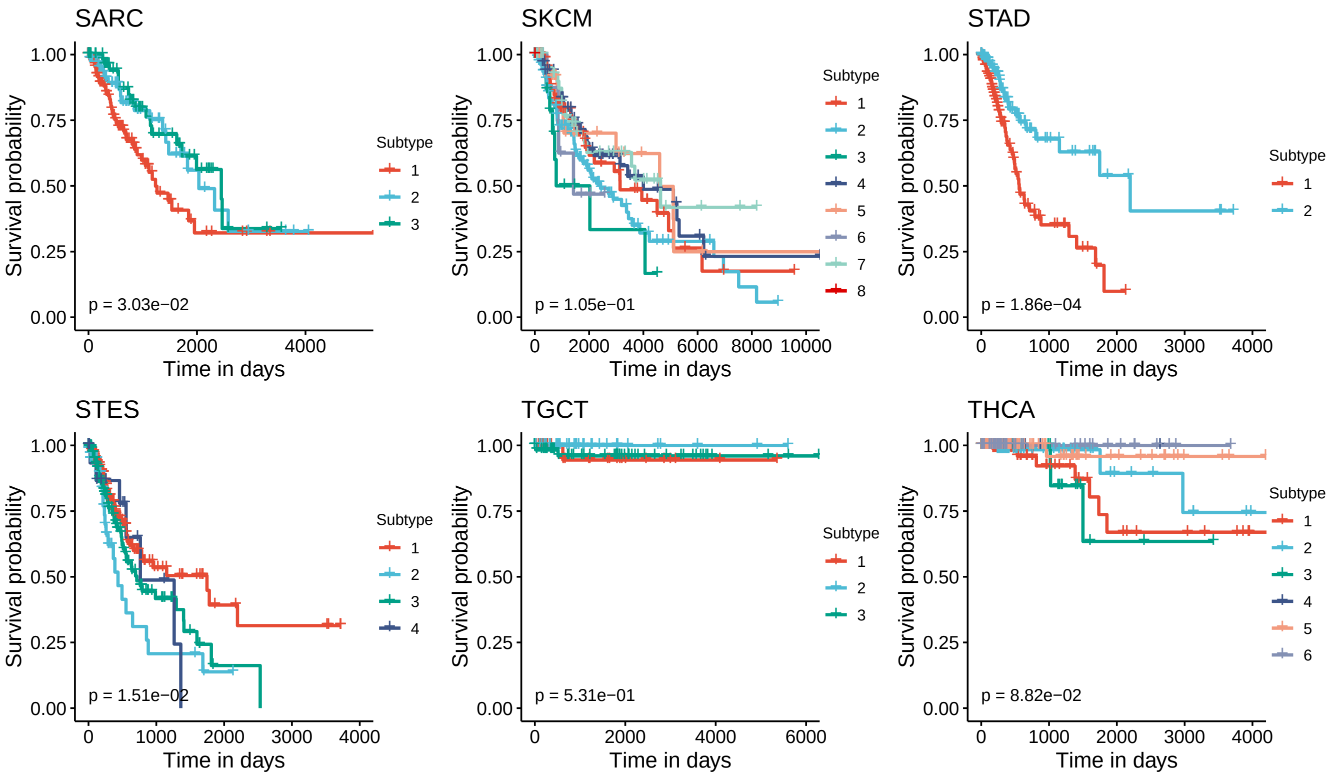

Figure S14. Kaplan-Meier survival analysis for TCGA-SARC, SKCM, STAD, STES, TGCT, and THCA datasets.

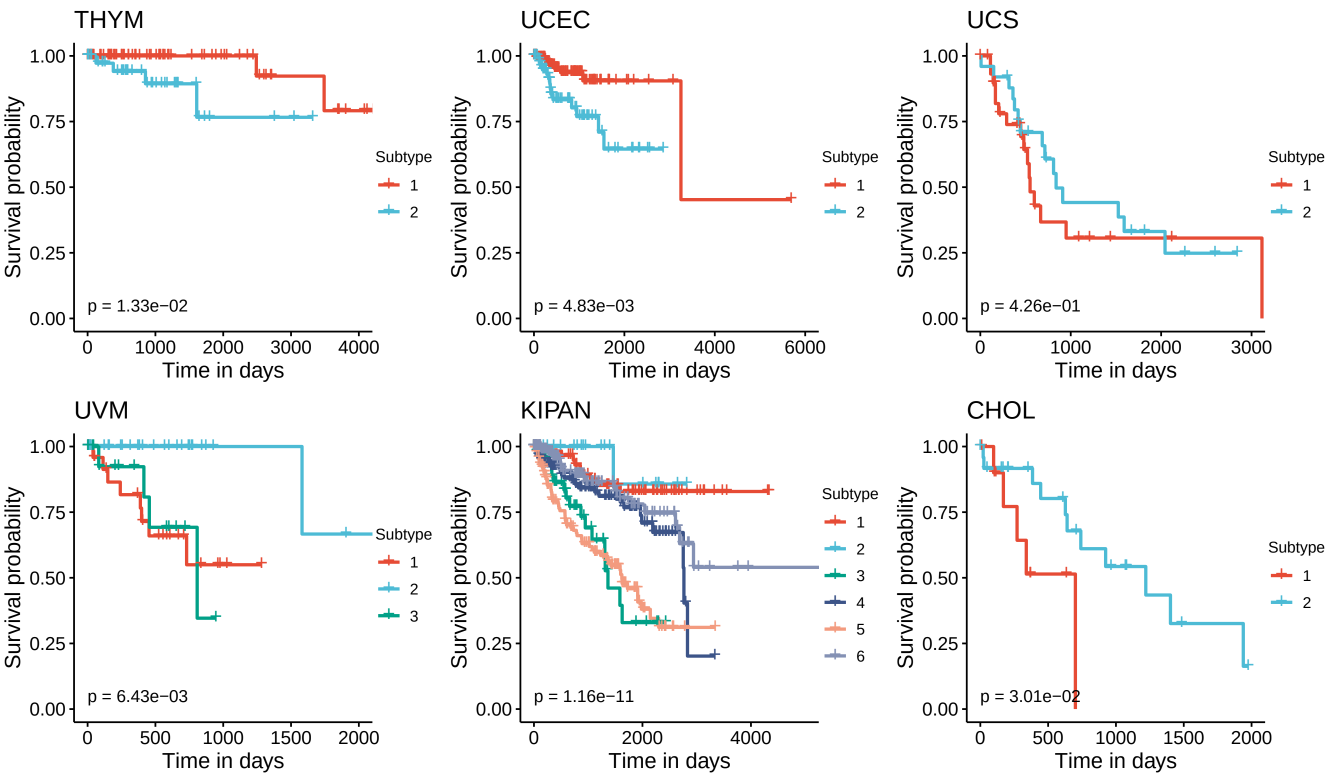

Figure S15. Kaplan-Meier survival analysis for TCGA-THYM, UCEC, UCS, UVM, KIPAN, and CHOL datasets.

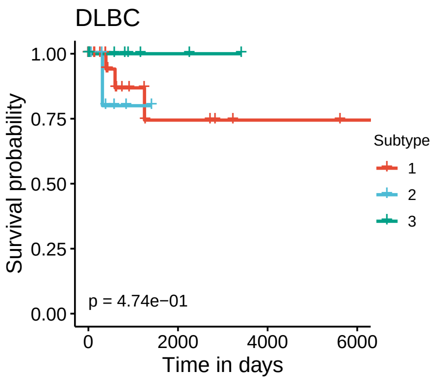

Figure S16. Kaplan-Meier survival analysis for TCGA-DLBC dataset.

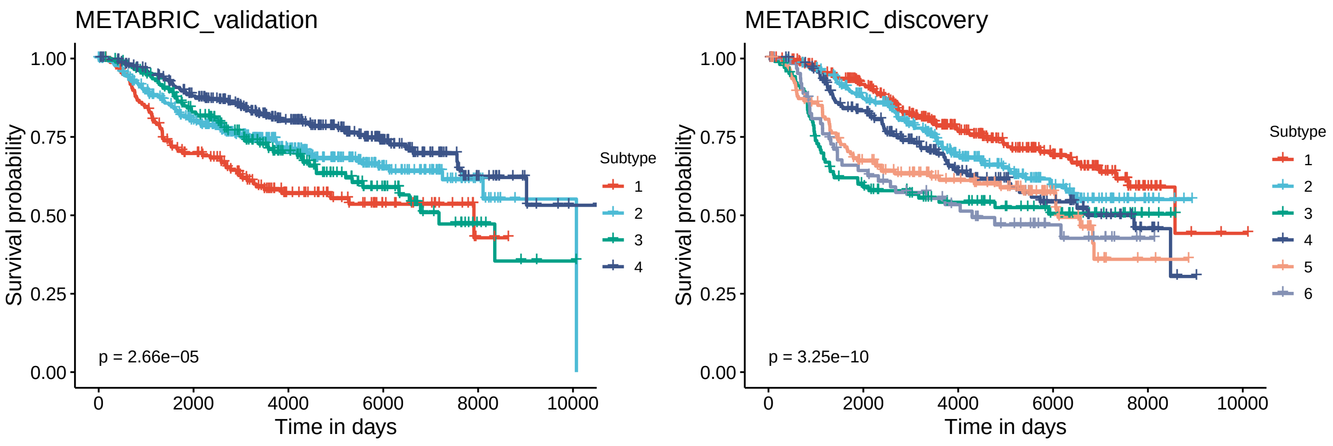

Figure S17. Kaplan-Meier survival analysis for METABRIC Validation and Discovery datasets.

10 CONTRIBUTION OF INDIVIVUAL OMIC TYPES

Table S15 reports the Cox-values obtained for each data type of the 37 TCGA datasets. The data shows that mRNA plays a very important role in subtyping ACC, BLCA, LAML, MESO, PAAD, SARC, and SKCM datasets. In these cancers, subtypes discovered from mRNA data have more significant Cox p-values than those from other data types (methylation and miRNA). For BLCA, LAML, SARC, and SKCM, mRNA is the only data type for which SMRT can discover subtypes with significant survival differences. The second data type, DNA methylation, is very important for CHOL, GBM, GBMLGG, KICH, KIRP, LGG, THYM, and UCEC. Subtyping using methylation yields more significant Cox p-values than mRNA and miRNA. In fact, methylation is the only data type that provides significant Cox p-values among the three data types for CHOL, KICH, and UCEC. The third data type, miRNA, is important for BRCA, CESC, COAD, COADREAD, KIPAN, PAAD, READ, STES, and UVM. The Cox p-values of miRNA are more significant than those of mRNA and methylation in these datasets.

While each data type contributes differently to the integrated subtypes in each dataset, it is clear that the numbers of datasets with significant p-values for individual data types are substantially smaller than that obtained from data integration. These numbers are 12, 14, and 13 for mRNA, methylation, and miRNA, respectively, compared to 26 for data integration. More importantly, the p-values obtained from data integration are more significant in most of those datasets (20 out of the 26 significant datasets). In some datasets (e.g., HNSC, KIRC, LUSC), none of the data types provide sufficient information to determine subtypes with significantly different survivals. However, when we integrate these data types, SMRT is able to exploit the complementary information available in each data type to determine subtypes with significant survival differences.

Table S15. Cox p-values of clustering results by SMRT for each data type of 37 TCGA datasets.

Dataset	mRNA	Methylation	miRNA	Integration
1. ACC	3.55e-03	2.81e-02	3.81e-01	1.33e-02
2. BLCA	1.95e-02	7.11e-02	7.30e-02	1.95e-02
3. BRCA	3.81e-01	4.02e-01	1.96e-03	1.96e-03
4. CESC	2.90e-01	4.88e-02	2.95e-02	2.95e-02
5. CHOL	9.62e-01	3.01e-02	5.56e-01	3.01e-02
6. COAD	6.13e-01	3.05e-01	1.44e-03	1.44e-03
7. COADREAD	5.82e-01	8.68e-01	2.89e-03	2.89e-03
8. DLBC	7.28e-01	4.12e-01	9.37e-01	4.74e-01
9. ESCA	2.58e-01	5.27e-01	3.92e-01	3.30e-01
10. GBM	4.08e-01	1.25e-04	5.19e-02	8.75e-05
11. GBMLGG	2.16e-13	3.29e-16	8.26e-03	7.48e-17
12. HNSC	2.84e-01	5.83e-01	2.61e-01	4.56e-02
13. KICH	1.88e-01	1.02e-04	1.88e-01	2.77e-02
14. KIPAN	2.58e-06	4.25e-02	1.16e-07	1.16e-11
15. KIRC	1.76e-01	1.11e-01	1.38e-01	5.98e-05
16. KIRP	2.38e-03	1.24e-05	4.45e-02	1.15e-09
17. LAML	3.47e-03	4.42e-01	7.24e-02	8.72e-04
18. LGG	1.13e-04	3.29e-16	8.88e-16	4.26e-15
19. LIHC	2.62e-01	2.86e-01	6.83e-01	7.04e-01
20. LUAD	1.25e-01	8.49e-01	4.16e-01	4.66e-01
21. LUSC	1.25e-01	1.57e-01	1.17e-01	8.37e-03
22. MESO	6.69e-03	2.05e-02	1.96e-02	7.34e-04
23. OV	8.01e-01	1.22e-01	6.76e-01	6.81e-01
24. PAAD	6.91e-04	1.44e-03	6.91e-04	2.73e-04
25. PCPG	7.44e-01	8.66e-01	4.09e-01	8.66e-01
26. PRAD	4.97e-01	7.25e-01	8.93e-01	3.49e-01
27. READ	6.49e-01	6.27e-01	8.37e-03	2.35e-02
28. SARC	4.06e-02	8.17e-02	7.35e-01	3.03e-02
29. SKCM	5.16e-03	7.69e-01	1.78e-01	1.05e-01
30. STAD	8.43e-01	4.12e-01	3.71e-01	1.86e-04
31. STES	3.78e-01	1.66e-01	1.82e-02	1.51e-02
32. TGCT	3.89e-01	5.31e-01	5.90e-01	5.31e-01
33. THCA	5.59e-01	1.26e-01	4.93e-01	8.82e-02
34. THYM	1.87e-02	5.60e-03	1.87e-01	1.33e-02
35. UCEC	2.12e-01	4.83e-03	5.92e-01	4.83e-03
36. UCS	8.34e-01	8.13e-01	4.26e-01	4.26e-01
37. UVM	3.37e-01	2.53e-03	5.69e-04	6.43e-03

REFERENCES

Ali, I. U., Schriml, L. M., and Dean, M. (1999). Mutational spectra of pten/mmac1 gene: a tumor suppressor with lipid phosphatase activity. *Journal of the National Cancer Institute* 91, 1922–1932

Cantanhede, I. G. and de Oliveira, J. R. M. (2017). Pdgf family expression in glioblastoma multiforme: data compilation from ivy glioblastoma atlas project database. *Scientific Reports* 7, 1–9

Carrasco-García, E., Saceda, M., and Martínez-Lacaci, I. (2014). Role of receptor tyrosine kinases and their ligands in glioblastoma. *Cells* 3, 199–235

Cenciarelli, C., Marei, H. E., Zonfrillo, M., Pierimarchi, P., Paldino, E., Casalbore, P., et al. (2014). Pdgf receptor alpha inhibition induces apoptosis in glioblastoma cancer stem cells refractory to anti-notch and anti-egfr treatment. *Molecular Cancer* 13, 1–15

Curtis, C., Shah, S. P., Chin, S.-F., Turashvili, G., Rueda, O. M., Dunning, M. J., et al. (2012). The genomic and transcriptomic architecture of 2,000 breast tumours reveals novel subgroups. *Nature* 486, 346–352

Gendoo, D. M., Ratanasirigulchai, N., Schroeder, M. S., Pare, L., Parker, J. S., Prat, A., et al. (2020). *genefu: Computation of Gene Expression-Based Signatures in Breast Cancer*. R package version 2.18.1

Hao, Z. and Guo, D. (2019). Egfr mutation: novel prognostic factor associated with immune infiltration in lower-grade glioma; an exploratory study. *BMC Cancer* 19, 1–13

Hubert, L. and Arabie, P. (1985). Comparing partitions. *Journal of Classification* 2, 193–218

Kaplan, E. L. and Meier, P. (1958). Nonparametric estimation from incomplete observations. *Journal of the American Statistical Association* 53, 457–481

Korotkevich, G., Sukhov, V., Budin, N., Shpak, B., Artyomov, M. N., and Sergushichev, A. (2021). Fast gene set enrichment analysis. *BioRxiv* , 060012

Louis, D. N., Perry, A., Reifenberger, G., Von Deimling, A., Figarella-Branger, D., Cavenee, W. K., et al. (2016). The 2016 world health organization classification of tumors of the central nervous system: a summary. *Acta Neuropathologica* 131, 803–820

Nguyen, H., Tran, D., Galazka, J. M., Costes, S. V., Beheshti, A., Draghici, S., et al. (2021). CPA: A web-based platform for consensus pathway analysis and interactive visualization. *Nucleic Acids Research* , gkab421

Ohgaki, H. and Kleihues, P. (2007). Genetic pathways to primary and secondary glioblastoma. *The American Journal of Pathology* 170, 1445–1453

Stupp, R., Hegi, M. E., Mason, W. P., van den Bent, M. J., Taphoorn, M. J., Janzer, R. C., et al. (2009). Effects of radiotherapy with concomitant and adjuvant temozolomide versus radiotherapy alone on survival in glioblastoma in a randomised phase iii study: 5-year analysis of the eortc-ncic trial. *The Lancet Oncology* 10, 459–466

Szerlip, N. J., Pedraza, A., Chakravarty, D., Azim, M., McGuire, J., Fang, Y., et al. (2012). Intratumoral heterogeneity of receptor tyrosine kinases egfr and pdgfra amplification in glioblastoma defines subpopulations with distinct growth factor response. *Proceedings of the National Academy of Sciences* 109, 3041–3046. doi:10.1073/pnas.1114033109

Verhaak, R. G. W., Hoadley, K. A., Purdom, E., Wang, V., Qi, Y., Wilkerson, M. D., et al. (2010). Integrated genomic analysis identifies clinically relevant subtypes of glioblastoma characterized by abnormalities in PDGFRA, IDH1, EGFR, and NF1. *Cancer Cell* 17, 98–110

Xu, G. and Li, J. Y. (2016). Differential expression of pdgfrb and egfr in microvascular proliferation in glioblastoma. *Tumor Biology* 37, 10577–10586

Yeo, A. T., Jun, H. J., Appleman, V. A., Zhang, P., Varma, H., Sarkaria, J. N., et al. (2021). Egfrviii tumorigenicity requires pdgfra co-signaling and reveals therapeutic vulnerabilities in glioblastoma. *Oncogene* 40, 2682–2696

Zhang, J. (2014). *CNTools: Convert segment data into a region by sample matrix to allow for other high level computational analyses*
